# Safety and Efficacy of Modular Digital Psychotherapy for Social Anxiety: Randomized Controlled Trial

**DOI:** 10.2196/64138

**Published:** 2025-04-10

**Authors:** Mona M Garvert, Jessica McFadyen, Stuart Linke, Tayla McCloud, Sofie S Meyer, Sandra Sobanska, Paul B Sharp, Alex Long, Quentin J M Huys, Mandana Ahmadi

**Affiliations:** 1 Alena London United Kingdom

**Keywords:** social anxiety disorder, randomized controlled trial, digital mental health, cognitive behavioral therapy, internet-delivered CBT

## Abstract

**Background:**

Social anxiety disorder is a common mental health condition characterized by an intense fear of social situations that can lead to significant impairment in daily life. Cognitive behavioral therapy (CBT) has been recognized as an effective treatment; however, access to therapists is limited, and the fear of interacting with therapists can delay treatment seeking. Furthermore, not all individuals respond. Tailoring modular treatments to individual cognitive profiles may improve efficacy. We developed a novel digital adaptation of CBT for social anxiety that is both modular and fully digital without a therapist in the loop and implemented it in the smartphone app Alena.

**Objective:**

This study aimed to evaluate the safety, acceptability, and efficacy of the new treatment in online participants with symptoms of social anxiety.

**Methods:**

In total, 2 web-based randomized controlled trials (RCTs) comparing individuals with access to the treatment through the app to a waitlist control group were conducted. Participants were recruited on the web and reported Social Phobia Inventory (SPIN) total scores of ≥30. Primary outcomes were safety and efficacy over 6 weeks in 102 women aged 18 to 35 years (RCT 1) and symptom reduction (SPIN scores) after 8 weeks in 248 men and women aged 18 to 75 years (RCT 2).

**Results:**

In RCT 1, active and control arm adverse event frequency and severity were not distinguishable (intervention: 7/52, 13%; waitlist control: 8/50, 16%; χ^2^_1_=0.007; *P*=.93). App acceptability was high, with a median completion rate of 90.91% (IQR 54.55%-100%). Secondary outcomes suggested greater symptom reduction in the active arm (mean SPIN score reduction −9.83, SD 12.80) than in the control arm (mean SPIN score reduction −4.13, SD 11.59; *t*_90_=−2.23; false discovery rate *P*=.04; Cohen *d*=0.47). RCT 2 replicated these findings. Adverse event frequency was comparable across the 2 groups (intervention: 20/124, 16.1%; waitlist control: 21/124, 16.8%; χ^2^_1_<0.001; *P*>.99). Despite a longer treatment program, median completion remained high (84.85%, IQR 51.52%-96.97%). SPIN score reduction was greater in the active arm (mean −12.89, SD 13.87) than in the control arm (mean −7.48, SD 12.24; *t*_227_=−3.13; false discovery rate *P*=.008; Cohen *d*=0.42).

**Conclusions:**

The web-only, modular social anxiety CBT program appeared safe, acceptable, and efficacious in 2 independent RCTs on online patient groups with self-reported symptoms of social anxiety.

**Trial Registration:**

ClinicalTrials.gov NCT05858294; https://clinicaltrials.gov/study/NCT05858294 (RCT 1) and ClinicalTrials.gov NCT05987969; https://clinicaltrials.gov/study/NCT05987969 (RCT 2)

## Introduction

Social anxiety disorder (SAD) is a prevalent and debilitating mental health challenge that affects a substantial portion of the global population at some point in their lives [1,2]. Characterized by a persistent fear of social situations, SAD can severely limit a person’s ability to engage in everyday activities, from forming personal relationships to navigating work and educational settings. In the long term, SAD can lead to profound social isolation; missed opportunities; and comorbid conditions such as depression, generalized anxiety disorder, and substance abuse [1,3,4].

While symptoms do not typically improve in the absence of treatment [5,6], cognitive behavioral therapy for social anxiety (CBT-SA) has been established as an effective treatment with moderate to large effects on social anxiety symptoms [7-9]. CBT-SA typically addresses the cognitive processes and behavioral patterns that sustain social anxiety, including negative self-perception in social interactions, self-directed attention, and anticipatory and postevent processing of social situations, and safety behaviors that, paradoxically, maintain anxiety because they prevent the disconfirmation of negative beliefs [10]. By challenging these patterns, cognitive behavioral therapy (CBT) facilitates significant improvements in symptoms, enabling individuals to engage more freely in social situations.

However, traditional CBT-SA faces limitations in accessibility [11] and effectiveness. A significant portion of those with SAD never seek treatment because interacting with a therapist, a cornerstone of traditional CBT, can be a phobic stimulus [12,13]. Moreover, in many places worldwide, the availability of trained therapists cannot meet the demand, leading to long wait times and further barriers to accessing care [14]. In response to these challenges, internet-delivered CBT (iCBT) has emerged as a promising alternative to traditional therapy [9,15-20]. These programs offer individuals the opportunity to work through therapeutic exercises and techniques at their own pace and provide discreet, affordable, and immediate support to those in need [21].

However, the quality and evidence base of existing applications vary significantly, and social anxiety apps often lack essential components such as interactive exercises and personalized feedback, limiting their effectiveness [22,23]. This highlights the need for robustly designed, theory-driven digital interventions that align with established therapeutic principles [24]. Furthermore, despite the increasing prevalence of digital interventions for SAD, limited attention has been paid to their potential risks or adverse events, such as increased anxiety or maladaptive coping. This gap is concerning as understanding the nature and frequency of adverse events is critical for ensuring the safety and acceptability of digital therapeutics. This study aimed to address this limitation by systematically documenting and analyzing adverse events reported during the trial, contributing to a more comprehensive understanding of the benefits and risks associated with digital interventions for social anxiety.

CBT-SA also does not always work and can be slow [25]. This may be because standard CBT involves a broad range of interventions aimed at various cognitive and behavioral processes, whereas individual patients may benefit predominantly from a specific subset of these interventions [26,27]. Hence, it may be possible to further improve treatment efficacy and speed by tailoring interventions to individual cognitive or behavioral profiles [28]. This requires breaking down CBT-SA into distinct, separable modules that target specific cognitive processes or mechanisms selectively [29]. Personalization of treatment may then be achieved by matching interventions to a person’s cognitive profile.

To address accessibility and work toward a modular targeted therapy, we developed a web-only, modular iCBT program based on the therapy by Clark and Wells [10]. In it, separate modules target each of the core cognitive components in the standard treatment, including negative beliefs, self-focused attention, rumination, and avoidance behaviors [30]. Challenging negative beliefs helps individuals develop more balanced views about themselves. Reducing self-focused attention shifts the attentional focus outward, lessening self-consciousness. Interrupting rumination limits the impact of perceived social failures, and reducing avoidance behaviors through exposure enables individuals to extinguish fear in social settings and correct cognitive biases. Each intervention can reduce the symptoms of social anxiety and promote more adaptive social functioning depending on an individual’s needs.

In this paper, we report the findings of 2 randomized controlled trials (RCTs) investigating the safety, acceptability, and efficacy of this iCBT program in the form of a smartphone app. The goal of RCT 1 was to establish the app’s safety, acceptability, and efficacy within a narrowly defined target group early in the development process. Modules were unlocked weekly to guide participants through a structured progression, and the trial was limited to young women (aged 18-35 years) to focus on a group with high social anxiety prevalence.

The goal of RCT 2 was to conceptually replicate the benefits of the core modules used in RCT 1 in the context of a broader sample and with improvements to the app’s adherence and engagement. Insights from RCT 1 then informed modifications tested in RCT 2, which involved a broader demographic (men and women aged 18-75 years) and adjustments aimed at optimizing engagement, accessibility, and efficacy. Specifically, insights from RCT 1 highlighted key barriers to engagement and adherence, particularly the structured weekly unlocking of modules, which some participants found restrictive. In response, RCT 2 adopted a more flexible approach allowing participants to access all modules from the start, enabling them to progress at their own pace. In addition, RCT 1 feedback suggested the need for enhanced engagement features. To address this, RCT 2 introduced a Community tab to foster connection and added Recharge exercises incorporating mindfulness and self-compassion components to support sustained reflection. Gamelike assessments were also integrated to encourage self-awareness and motivation aiming to further enhance adherence and overall efficacy. These modifications directly addressed challenges identified in RCT 1, ensuring a more accessible and engaging experience for participants.

Overall, the treatment remained safe, acceptable, and effective, significantly improving symptoms of social anxiety in 2 separate samples compared to a waitlist control group (RCT 1: n=102; RCT 2: n=267). This enabled the development of mechanistically defined cognitive assessment modules to personalize treatment delivery and, hopefully, further improve efficacy.

## Methods

### Objective

We aimed to examine whether the web-only, modular treatment program for social anxiety in the smartphone app was safe and acceptable in the first study and whether it was efficacious in reducing self-reported symptoms of social anxiety in the second study.

### Ethical Considerations

The studies received approval from the Reading Independent Ethics Committee (study reference: AYSATOL).

Participants provided informed consent digitally before engaging in any part of the study. They were presented with detailed participant information sheets outlining the study’s purpose and procedures and their rights, including their ability to withdraw at any time without penalty.

All study data were pseudonymized using Prolific IDs to ensure participant confidentiality. All data handling was compliant with the General Data Protection Regulation and UK Data Protection Act (2018). Data were securely stored and used only for the purposes stated in this study.

Engagement with the app and the therapeutic content itself was not incentivized. However, participants received £1 (US $1.27) for their involvement in the screening process. Furthermore, all participants in both RCTs were compensated with £5 (US $6.36) per survey independently of engagement with the app or therapy.

### Clinical Trial Registration

RCT 1 was retrospectively registered on ClinicalTrials.gov after data collection was completed (NCT05858294). For RCT 2, statistical analyses were preregistered on ClinicalTrials.gov before data collection began (NCT05987969).

### Design

We conducted 2 web-based, unblinded RCTs. RCT 1 was a 6-week parallel-group RCT with a 4-week intervention and a 2-week follow-up. RCT 2 was an 8-week trial with a 4-week follow-up. In both trials, participants were randomized 1:1 to the active arm with access to the smartphone app or the control arm without access to the smartphone app. In RCT 1, participants in the control arm were given access to the smartphone app at week 4. In RCT 2, they were given access to the app at week 12. The randomization algorithm used to assign participants to the treatment and control groups was set up by a technical support team that was not involved in data analysis or outcome assessment.

### Outcomes

The primary outcome measures in RCT 1 were safety and acceptability. Secondary outcome measures were symptoms and functioning at week 4 and 2 weeks after the intervention at week 6.

The primary outcome measures in RCT 2 were change in symptoms and daily functioning from baseline to week 8. Secondary outcome measures were safety, efficacy, and daily functioning 4 weeks after the end of the intervention at week 12.

Safety was monitored through items in the weekly surveys that asked participants to report any new serious adverse effects experienced in the previous week. The intervention group was asked the following: “Have you experienced any negative effects from using the Alena app? This could be a physical or emotional effect that you believe you have experienced as a result of using the app and/or engaging in the app therapy.” Both groups were asked the following: “Have you experienced any new, serious negative health effects in the past week? This includes having to see your GP for a new reason, going to hospital, or being otherwise very unwell in terms of your physical or mental health.” If participants responded positively to either question, they were prompted for additional details and to rate the severity of the event. Any reported events were reviewed by a clinician, who determined whether the effect matched criteria for a *serious adverse event* as defined by the International Organization for Standardization 14155 standard. Due to a technical problem, adverse events were not recorded for the first week in the waitlist control group. This suggests that the total number of adverse events experienced by the waitlist control group but not the intervention group may have been underestimated.

Acceptability was assessed using custom-built questionnaires. Participants were asked how satisfied they were with the app overall (5-point Likert scale from very dissatisfied to very satisfied); how helpful they found the app (5-point Likert scale from very unhelpful to very helpful); how likely they would be to recommend the app (5-point Likert scale from very unlikely to very likely); how easy they found using the app (5-point Likert scale from very difficult to very easy); whether they got to the end of the weekly exercise (yes or no); and what got in the way of completing the exercises, with options provided. Furthermore, adherence to the therapy (monitored using in-app event markers) was monitored through participants’ engagement with the app.

Symptoms were measured using the Social Phobia Inventory (SPIN) [31]. Designed to evaluate the comprehensive range of symptoms associated with social anxiety—such as fear, avoidance, and physiological reactions—the SPIN includes 17 items, each scored from 0 to 4. This scoring system yields a total possible score ranging from 0 to 68. A score of >19 separates individuals with social anxiety from controls without anxiety [31,32]. A decrease of ≥10 points from the baseline SPIN score is considered a reliable indicator of significant improvement in social anxiety according to the Reliable Change Index provided by the National Collaborating Centre for Mental Health (2018). A score of ≤19 corresponds to subclinical levels of anxiety. The SPIN has demonstrated strong internal consistency, with Cronbach α coefficients ranging from 0.87 to 0.94 in patients with social anxiety and 0.82 to 0.90 in controls [31].

Daily functioning was assessed using the Work and Social Adjustment Scale (WSAS) [33]. The WSAS evaluates how much a respondent’s issue affects their ability to perform everyday tasks, including work, managing home responsibilities, and engaging in social and leisure activities. Each activity is rated on a scale ranging from 0 (“not at all”) to 8 (“very severely”), with total scores ranging from 0 to 40. The WSAS exhibits good internal consistency, with Cronbach α values between 0.70 and 0.94 [33].

All outcome assessments, including follow-up surveys, were self-reported and conducted on the web via the Prolific platform. The research team analyzing the primary outcomes (SPIN and WSAS scores) was not blinded. A clinician blinded to group assignments reviewed and rated participant reports of adverse events to ensure an unbiased assessment.

### Eligibility Criteria

The inclusion criteria were as follows:

1. Social anxiety symptom severity: SPIN total score of ≥30, indicating a moderate to severe level of social anxiety
2. Stability of mental health medication: unchanged dose for ≥8 weeks
3. Age between 18 and 35 years for RCT 1 and between 18 and 75 years for RCT 2
4. Female sex (RCT 1 only)
5. Smartphone with iOS and internet access for RCT 1 and smartphone with internet access and Android or iOS operating system for RCT 2

The exclusion criteria for both studies were as follows:

1. Alcohol use was assessed with the Alcohol Use Disorders Identification Test for Consumption [34] to determine the risk of alcohol dependence. Eligibility required participants to report less than a severe risk level (<8 points out of a possible 12).
2. Participants were screened for recreational drug use using the following three questions—(1) “Have you used any recreational drugs in the last three months?” (2) “In the last three months, have you had a strong desire or urge to use recreational drugs at least once a week or more often?” (3) “In the last three months, has anyone expressed concern about your use of recreational drugs?” Eligibility was limited to those reporting minimal to no use (<2 points out of a possible 3).
3. Participants who had previously used the Alena app were excluded.

### Recruitment

All interactions and data collection occurred on the web via Prolific, an online recruitment platform. Participants initially underwent a screening process using a web-based questionnaire that collected information on demographics, lifestyle habits, mental health history, and access to technology. If they passed the screening, they were offered participation in the RCTs.

### Intervention

**Figure 1:**
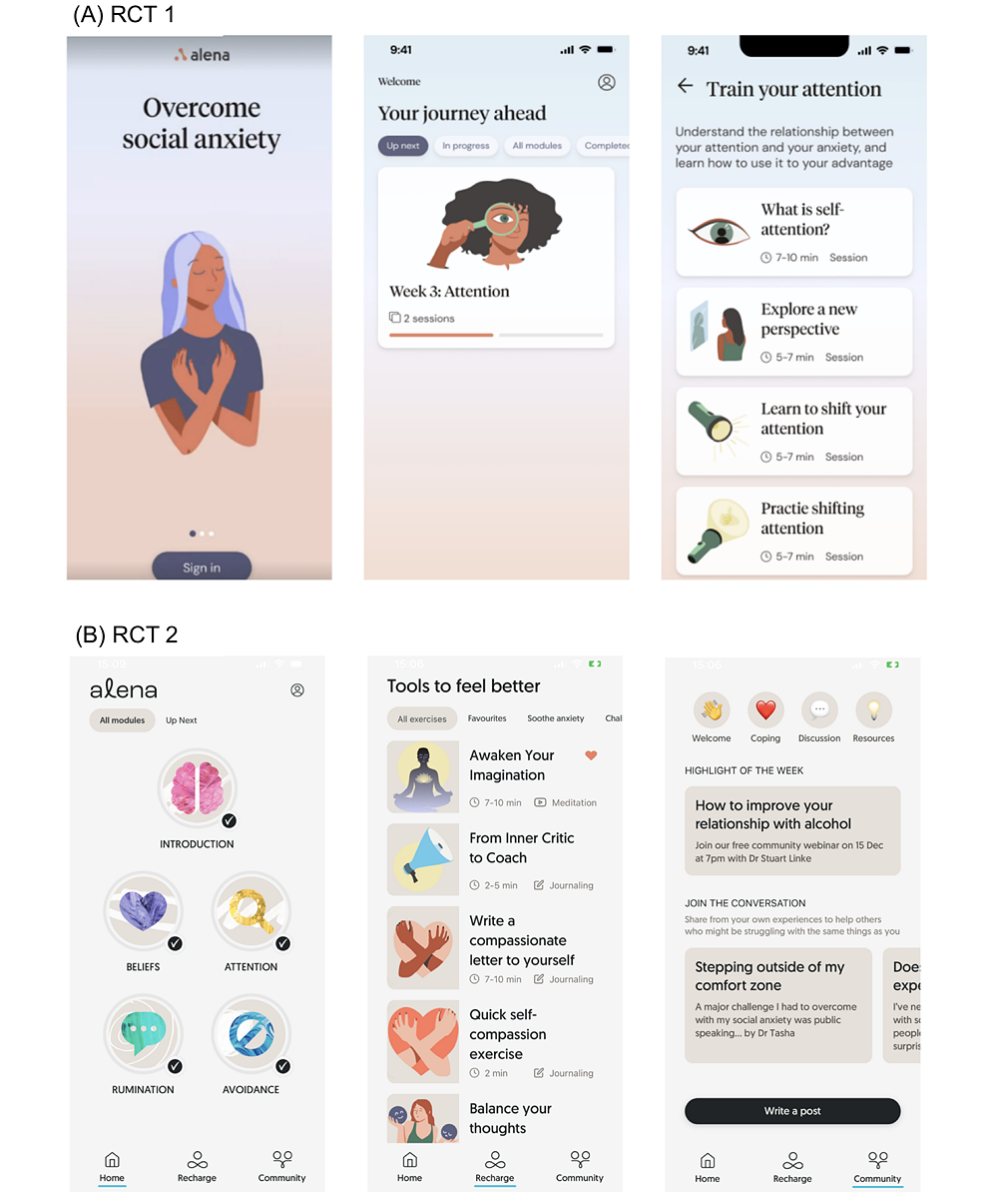
User interface of the apps used in the 2 randomized controlled trials (RCTs). (A) Interfaces used in RCT 1: the sign-in screen (left), program overview with filters for displaying different modules (middle), and a list of exercises for a particular module (eg, attention; right). (B) Updated user interface used in RCT 2: the home screen showing each module, which could be tapped to show a list of exercises (left); the Recharge screen showing a list of exercises not included in the main program but still centered on alleviating social anxiety (middle); and a Community screen showing forum posts from members of the Alena community (right).

The treatment consisted of access to the smartphone app (Figures 1A and B). The program was designed in line with the CBT competencies framework [35] and consisted of an introductory module focusing on psychoeducation as well as 4 modules each targeting a key mechanism of SAD (see Multimedia Appendix 1 for a detailed program outline): (1) the Introduction module served as an introductory overview, setting the stage for the program and providing insights into the drivers of social anxiety symptoms; (2) the Beliefs module focused on conditional beliefs about oneself and others; (3) the Attention module concentrated on self-awareness and self-focus during social interactions; (4) the Avoidance module dealt with safety behaviors and avoidance patterns; and (5) the Rumination module addressed the tendency to overthink or analyze social interactions after they occur.

Each module contained psychoeducational audio lessons and practical worksheets to guide participants through the content (Multimedia Appendix 1). In RCT 2, we updated the visual design and introduced more therapeutic content, including gamelike assessments to engage participants further and assess their cognitive and behavioral patterns related to social anxiety. These assessments, lasting between 5 and 15 minutes, were positioned at the start of each module, and completion was required to unlock the rest of the exercises within that module. In RCT 2, participants also had access to the Alena social anxiety community as well as Recharge exercises, such as brief meditation and compassion exercises.

### Recharge and Community Tabs

#### Overview

Participants in RCT 2 additionally had access to a Recharge and a Community section on the app (Figure 1B). The Recharge section contained mindfulness-based exercises, guided meditations, journaling, and self-compassion exercises designed to help participants overcome negative thoughts and feelings related to social situations. For example, participants learned how to observe thoughts without automatically identifying with them, wrote a compassionate letter to themselves, and were encouraged to celebrate a small win. The anonymous community provided an opportunity to connect with and receive support from others by posting in an online forum.

The community was moderated by a member of our team, who ensured that the content posted was appropriate, supportive, and relevant to social anxiety. This included reviewing posts for appropriateness before publication, discouraging harmful language, and intervening if discussions became triggering or distressing for other users. The moderators also actively prompted discussion relevant to social anxiety by posting relevant content. Critically, they did not offer any direct therapeutic advice. They responded with supportive comments or resources if a participant expressed distress. Participants were suggested to try 1 Recharge exercise per week and engage with a Community post in some way each week (by liking or commenting and considering posting if they felt comfortable).

#### Program Pacing

The exercises, each taking between 1 and 8 minutes to complete, were designed to fit into the users’ daily routine. The app encouraged participants to repeat exercises if needed and extend their learning outside the app through real-life exposure experiments supported by in-app exercises that assisted participants with planning and reflecting on these experiments.

To optimize the learning curve and ensure a structured progression through the program, the availability of modules was controlled. In RCT 1, modules were sequentially unlocked each week, whereas in RCT 2, all modules were accessible from the start, but participants were advised to complete 1 module every 2 weeks. To complete all recommended content on the app, participants would have needed to spend between 10 and 20 minutes on the app per week.

### Procedure

Following screening, participants underwent baseline assessments, including the SPIN, the WSAS, demographics, treatment expectations, and previous experience with mental health apps. Participants were then informed of their group assignment. Those in the intervention group received instructions on downloading and using the Alena app.

Participants in the waitlist control condition were informed not to access the publicly available app until the study period ended. Study app access was controlled via unique IDs, and use monitoring confirmed no unauthorized access. Use of the publicly available Alena app or other mental health apps during the study was not actively monitored and, therefore, cannot be ruled out.

During the intervention or waitlist phase, participants completed the SPIN and WSAS measures every week. Those using the app answered additional questions about their app use. After the intervention phase, app access was withdrawn from the initial intervention group. A follow-up survey was conducted 2 weeks later in RCT 1 and 4 weeks later in RCT 2 to assess short-term maintenance and collect final participant feedback.

### Power Calculations

We based our a priori effect size estimate on findings of previous digital-only CBT treatments for social anxiety [36], which demonstrated an effect size of *d*=0.67. To ensure a conservative approach, we rounded this estimate down to *d*=0.6. On the basis of a G*Power analysis for the difference between the groups (2-tailed, 2-sided *t* test), with an estimated medium effect size (Cohen *d*) of 0.6, an α level of .05, and 80% power, the required sample size was 45 participants per group. Considering a 10% likelihood of participant dropout, we increased our target sample size to 50 participants per group.

Sample sizes for RCT 2 were based on effect size estimates from RCT 1 (Cohen *d* of 0.47 after 4 weeks). To detect effect sizes of 0.47 with an α level of .05 and a power of 95%, a sample size of 119 participants per group was required. Considering the likelihood of participant dropout, we increased our target sample size to 125 participants per group.

### Statistical Analyses

#### Comparison of Baseline Characteristics Between the Groups

We conducted Bayesian analyses in JASP (0.18.3) to assess evidence for a null hypothesis that both groups were the same, quantified using the Bayes factor for the null hypothesis (BF_01_). Bayesian analysis provides a probabilistic framework that allows for direct comparisons of hypotheses. Specifically, unlike traditional frequentist methods, Bayesian analysis can quantify the strength of evidence for both the null and alternative hypotheses. If BF_01_≥3, this indicates evidence for the null hypothesis, whereas a value of <1 indicates evidence for the alternative hypothesis (that the groups are different). A value between 1 and 3 indicates insufficient evidence for either hypothesis. For continuous variables, we implemented Bayesian independent-sample *t* tests [37], and for categorical or binary variables, we implemented Bayesian contingency tables using an independent multinomial sampling method (groups fixed [6]).

#### Safety

We compared the number of adverse events between groups using a chi-square test.

#### Acceptability

We characterized acceptability descriptively. Dropout rates were also compared using a chi-square test.

#### Efficacy and Daily Functioning

Planned intention-to-treat analyses were performed to compare SPIN (efficacy) and WSAS (daily functioning) scores across groups. Specifically, we used independent *t* tests to compare mean changes in scores from baseline to the end of the intervention period and from the end of the intervention period to the follow-up between the intervention and waitlist control groups. We used the Benjamini-Hochberg method of false discovery rate (FDR) correction to correct for multiple comparisons. Chi-square tests were used to analyze categorical outcomes across groups, such as the proportion of participants who achieved a clinically significant improvement in social anxiety symptoms (defined as a reduction of ≥10 points in SPIN scores) and those who reached subclinical levels of symptoms (SPIN score of ≤19).

In an exploratory analysis, linear mixed-effects regression modeling was implemented to evaluate the change in SPIN and WSAS scores over time. The models included fixed effects for age, group, and time (week); a quadratic time effect (week^2^) accounting for nonlinear change in scores over time such as plateau effects; and a group × time interaction to assess differential changes in scores between the intervention and control groups throughout the study period. In RCT 2, additional fixed effects included sex, a group × sex, and a group × week × sex interaction to examine differential changes in scores by sex. Furthermore, the model included a random intercept for each participant, accounting for the baseline variability in scores among individuals.

The full model outputs, including all coefficients, SDs, and *P* values, are provided in Tables S1 and S2 in Multimedia Appendix 1.

All analyses were conducted using Python (version 3.8.9; Python Software Foundation) and R (version 4.4.0; R Foundation for Statistical Computing).

## Results

### Participants

**Figure 2:**
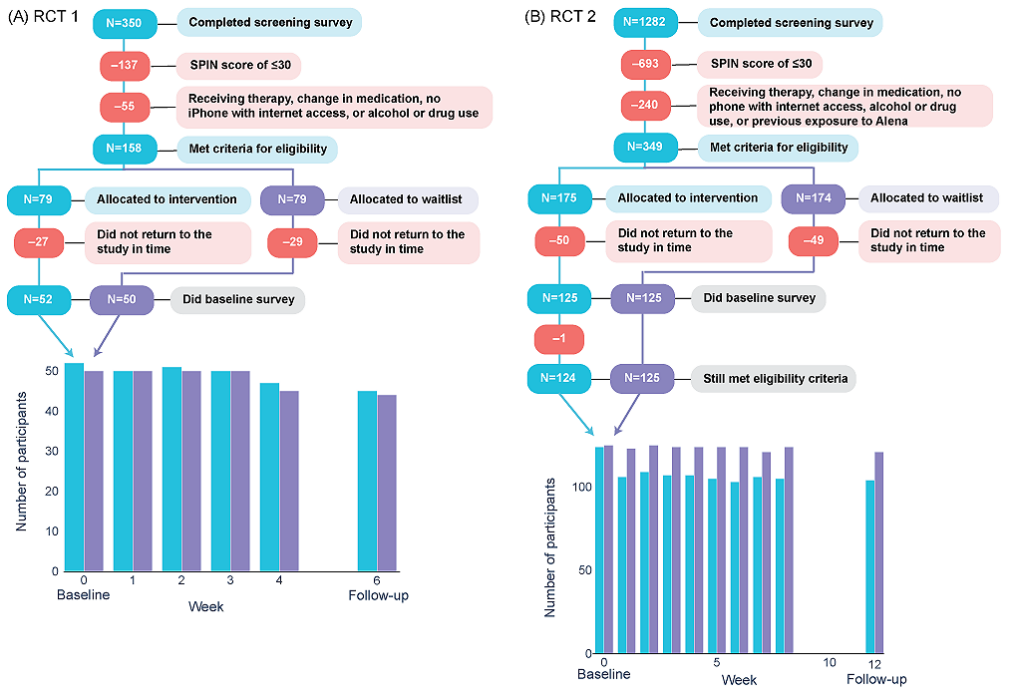
CONSORT (Consolidated Standards of Reporting Trials) diagram. Flow of participants through the study for randomized controlled trial (RCT) 1 (A) and RCT 2 (B). The number of participants who completed the questionnaires at each time point is visualized at the bottom. SPIN: Social Phobia Inventory.

Eligible participants from the screening studies (158/350, 45.1% in RCT 1 and 349/1282, 27.22% in RCT 2; Figure 2) were invited to take part in the main study. RCT 1 included a total of 102 participants (all female aged between 18 and 35 years; n=52, 51% in the intervention group and n=50, 49% in the control group; Figure 2A). RCT 2 included 249 participants in total (n=159, 63.9% female and n=90, 36.1% male aged between 18 and 75 years; Figure 2B). One participant in the intervention group of RCT 2 was excluded from the study due to reporting that they no longer had access to a smartphone with internet access in the baseline assessment, yielding 124 participants in the intervention group and 124 in the control group (Multimedia Appendix 2).

The groups in RCT 1 were equivalent on all measures (Table 1) except for age—the intervention group was 1.66 years older on average (mean age 29.12, SD 4.07 y) compared to the waitlist control group (mean age 27.46, SD 4.61 y; BF_01_=0.933, Bayesian independent-sample *t* tests). The groups in RCT 2 were balanced on all baseline characteristics (Table 1).

**Table 1:** Baseline characteristics of the study participants^a^.

Characteristic			RCT^b^ 1								RCT 2						
			Intervention (n=52)		Waitlist control (n=50)		BF_01_^c^			Intervention (n=124)			Waitlist control (n=124)			BF_01_	
Age (y), mean (SD)			29.12 (4.07)		27.46 (4.61)		0.933^d^			39.37 (10.53)			38.15 (10.84)			4.910^e^	
SPIN^f^ score, mean (SD)			43.81 (9.14)		43.28 (7.59)		4.575^e^			44.54 (8.35)			43.96 (9.34)			6.348^e^	
WSAS^g^ score, mean (SD)			18.63 (8.27)		19.52 (6.66)		4.087^e^			19.04 (7.21)			19.1 (8.02)			7.192^e^	
Expectations for Alena (score of 1-5), mean (SD)			2.37 (0.69)		2.28 (0.73)		4.055^e^			2.27 (0.7)			2.24 (0.72)			6.730^e^	
**Ethnicity, n (%)**								37.312^e^									>–100^e^
	Asian	1 (1.9)		3 (6)					6 (4.8)			8 (6.5)					
	Black	2 (3.8)		2 (4)					6 (4.8)			1 (0.8)					
	Mixed or multiple	6 (11.5)		2 (4)					6 (4.8)			3 (2.4)					
	White	43 (82.7)		43 (86)					103 (83.1)			111 (89.5)					
	Other	0 (0)		0 (0)					3 (2.4)			2 (1.6)					
**Employment status, n (%)**								>–100^e^									>–100^e^
	Full time	34 (65.4)		32 (64)					66 (53.2)			62 (50)					
	Part time	8 (15.4)		8 (16)					24 (19.4)			27 (21.8)					
	Student	5 (9.6)		7 (14)					7 (5.6)			5 (4)					
	Retired	0 (0)		0 (0)					2 (1.6)			2 (1.6)					
	Unemployed	2 (3.8)		2 (4)					13 (10.5)			15 (12.1)					
	Unable to work	1 (1.9)		1 (2)					6 (4.8)			5 (4)					
	Temporarily not working	2 (3.8)		0 (0)					6 (4.8)			9 (7.3)					
**Educational level, n (%)**								14.507^e^									>–100^e^
	No qualifications	0 (0)		0 (0)					1 (0.8)			1 (0.8)					
	GCSE^h^ or equivalent	2 (3.8)		2 (4)					16 (12.9)			15 (12.1)					
	A-level or equivalent	10 (19.2)		17 (34)					18 (14.5)			30 (24.2)					
	Apprenticeship, higher education diploma, or equivalent	4 (7.7)		4 (8)					14 (11.3)			8 (6.5)					
	Bachelor’s degree or equivalent	36 (69.2)		27 (54)					49 (39.5)			54 (43.5)					
	Postgraduate degree or equivalent	0 (0)		0 (0)					24 (19.4)			14 (11.3)					
	PhD or equivalent	0 (0)		0 (0)					2 (1.6)			3 (2.4)					
Alcohol use (AUDIT-C^i^ score), mean (SD)			2.71 (1.71)		2.48 (1.74)		3.898^e^			2.4 (1.97)			2.02 (2.16)			2.685^e^	
Any drug use, n (%)			5 (9.6)		2 (4)		4.524^e^			3 (2.4)			6 (4.8)			10.461^e^	
Ever had therapy, n (%)			44 (84.6)		35 (70)		1.069^j^			53 (42.7)			50 (40.3)			5.860^e^	
Ever had therapy—no but currently on a waitlist, n (%)			—^k^		—		—			12 (9.7)			17 (13.7)			6.260^e^	
On medication, n (%)			12 (23.1)		9 (18)		4.180^e^			27 (21.8)			27 (21.8)			7.688^e^	
Used apps for mental health before, n (%)			28 (53.8)		22 (44)		2.534^j^			38 (30.6)			42 (33.9)			6.009^e^	

^a^Group mean and SDs are shown for continuous variables (eg, age), and the number of participants and group percentages are shown for categorical (eg, educational level) or binary (eg, any drug use) variables. We conducted Bayesian analyses to assess evidence for a null hypothesis that both groups were the same (Bayes factor for the null hypothesis). If the Bayes factor is of ≥3, this indicates evidence for the null hypothesis, whereas a value of <1 indicates evidence for the alternative hypothesis (that the groups are different). A value between 1 and 3 indicates insufficient evidence for either hypothesis.

^b^RCT: randomized controlled trial.

^c^BF_01_: Bayes factor.

^d^BF_01_<1; indicates evidence for the alternative hypothesis (that the groups were different).

^e^BF_01_≥ 3; indicates evidence for the null hypothesis (that both groups were the same).

^f^SPIN: Social Phobia Inventory.

^g^WSAS: Work and Social Adjustment Scale.

^h^GCSE: General Certificate of Secondary Education.

^i^AUDIT-C: Alcohol Use Disorders Identification Test for Consumption.

^j^BF_01_ between 1 and 3; indicates insufficient evidence for either hypothesis.

^k^Data not collected.

### Retention

A linear regression with retention as outcome revealed a significant effect of week (*P*=.004) but not group (*P*=.47) for RCT 1, reflecting a decrease in retention over time in both groups. In RCT 2, there was a significant effect of group (*P*<.001) but not week (*P*=.43), with better retention for the waitlist control group. The week × group interaction was not significant in either RCT (*P*>.10 in both cases), suggesting that retention over time was not affected by group.

### Safety

In RCT 1, a total of 13% (7/52) of the participants allocated to the intervention group and 16% (8/50) of the participants allocated to the waitlist control group reported adverse effects at some point during the study (χ^2^_1_=0.007, *P*=.93; Multimedia Appendix 1). In total, fewer negative health effects were reported in the intervention group, although this difference was not significant (intervention: 9/52, 17% of reports; waitlist: 14/50, 28% of reports; χ^2^_1_=0.9, *P*=.34).

In RCT 2, a total of 16.1% (20/124) of the participants allocated to the intervention group and 16.8% (21/124) of the participants allocated to the waitlist control group reported experiencing adverse effects at some point during the study (χ^2^_1_<0.001, *P*>.99). In total, an equivalent number of negative health effects were reported between the groups (intervention: 31/124, 25% of reports; waitlist: 30/124, 24.2% of reports; χ^2^_1_=0.001, *P*=.98).

Most of the adverse events reported by the intervention groups in both RCTs were rated as mild or very mild. Only 1 adverse event in RCT 1 was rated as serious (“I got covid for the first time and I was hospitalised because of it. I was exhausted and in pain all week.”) but judged to be unrelated to the intervention. The events judged by participants in the intervention group as being related to using the Alena app were mild to moderate in severity and in line with what would be expected for a psychological therapy, where encountering anxiety-inducing situations in a controlled manner is essential for treatment effectiveness. No severe or very severe negative effects were reported from using the Alena app during the trial (a complete list of adverse events reported by participants is provided in Supplementary Material S3 in Multimedia Appendix 1).

### Acceptability

To assess user satisfaction and perceived utility, we collected subjective ratings from the intervention groups in both RCTs on various aspects of their experience using the app each week. These aspects included overall satisfaction with the app, its perceived helpfulness, the ease of use, and the likelihood of recommending the app to others.

**Figure 3:**
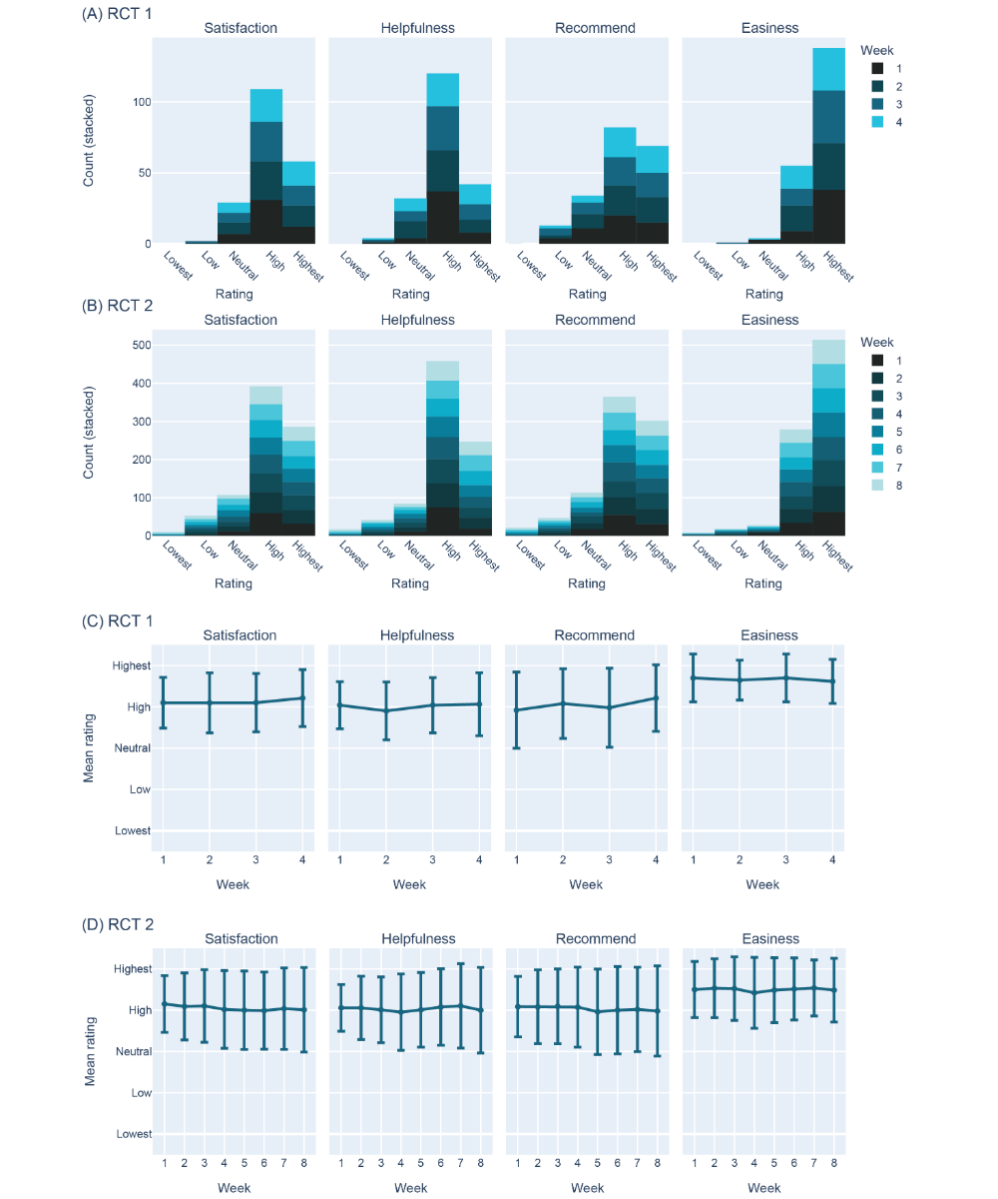
Acceptability ratings of the Alena app. We measured acceptability in 4 categories: how satisfied participants were with the app, how helpful they found the app, how likely they were to recommend the app, and how easy the app was to use. Response options ranged from 1 (lowest) to 5 (highest). Measures were taken each week (see the legend for the color scale) in both randomized controlled trial (RCT) 1 (A) and RCT 2 (B). Panels C and D visualize average ratings across weeks. Error bars denote the SD.

The feedback from participants in both RCT 1 and RCT 2 consistently reflected high levels of acceptability (Figures 3A-3D). Participants rated the app highly across all measures, with median ratings reaching 4 out of 5 for satisfaction (both RCTs IQR 4-5), helpfulness (RCT1 IQR 4-4, RCT 2 IQR 4-5), and likelihood of recommendation (both RCTs IQR: 4-5) and the maximum of 5 out of 5 for ease of use (both RCTs IQR 4-5). Overall, these findings suggest that the Alena app was highly acceptable to participants.

### Therapy Adherence

**Figure 4:**
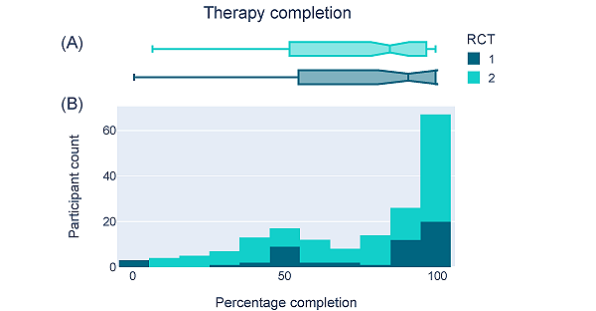
Therapy completion rates. (A) The box-and-whisker plot shows the distribution of therapy completion rates across participants in each RCT, with the median at the notch, the 25th to 75th percentiles represented by the box (ie, the IQR), and the whiskers of the plot representing each box boundary –1.5 to +1.5 × the IQR. (B) The histogram shows the proportion of exercises completed across participants in the intervention group for randomized controlled trial (RCT) 1 (dark blue) and RCT 2 (green).

Throughout the intervention period, we tracked how well participants adhered to Alena’s therapy program, monitoring the number of audio lessons listened to and interactive worksheets finished by each participant. Even though participants were not incentivized to adhere to the therapy program (they were only compensated for the time required to complete the weekly surveys), participants in RCT 1 showed a median completion rate of 90.91% (IQR 54.55%-100%; Figures 4A and 4B). For RCT 2, which featured a longer treatment program, the median completion rate was 84.85% (IQR 51.52%-96.97%; Figures 4A and 4B).

### Efficacy

**Figure 5:**
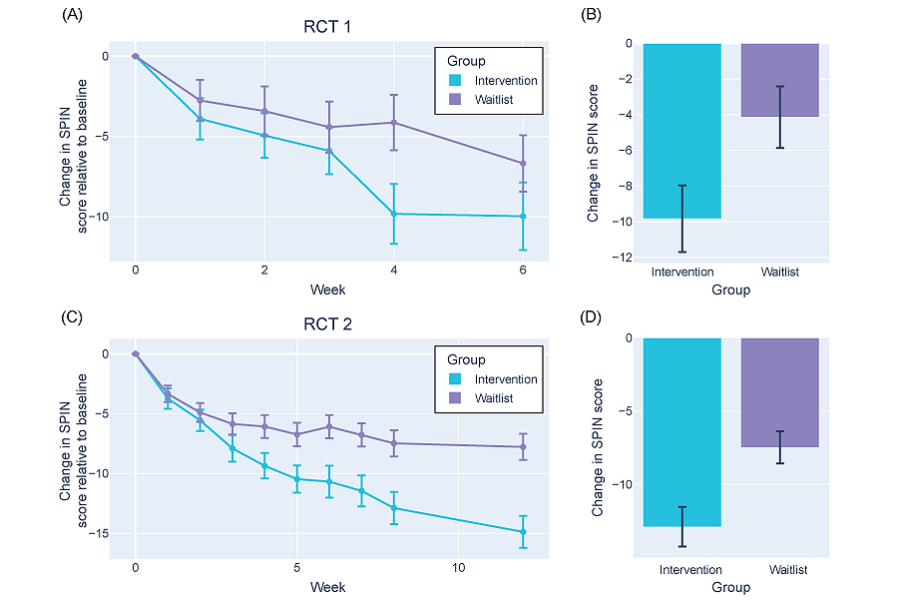
Improvement in social anxiety symptoms over time in both randomized controlled trials (RCTs). (A) Mean change in Social Phobia Inventory (SPIN) score across participants in either the intervention (blue) or the waitlist control (purple) group each week relative to the baseline assessment at week 0 in RCT 1. (B) Total change in SPIN score from week 0 to the final week of the intervention or waitlist period in RCT 1. (C) Mean change in SPIN score across participants in either the intervention (blue) or the waitlist control (purple) group each week relative to the baseline assessment at week 0 in RCT 2. (D) Total change in SPIN score from week 0 to the final week of the intervention or waitlist period in RCT 2. Error bars represent the SEM.

Participants in both RCTs had severe social anxiety symptoms at baseline. In RCT 1, the intervention group had a median SPIN score of 43 (IQR 35-51), whereas the waitlist control group had a median score of 42 (IQR 38-49; *t*_100_=0.32; *P*=.75). In RCT 2, the intervention group had a median SPIN score of 44 (IQR 39-50), and the waitlist control group had a median score of 41 (IQR 36-51; *t*_247_=0.52; *P*=.61). Symptom severity was tracked weekly using the SPIN (Figure 5).

By the end of the 4-week intervention period, participants in RCT 1 with access to the Alena app saw a significantly greater reduction in SPIN scores (mean −9.83, SD 12.80) compared to the waitlist control group (mean −4.13, SD 11.59; *t*_90_=−2.23; FDR *P*=.04; Cohen *d*=0.47; Figures 5A and 5B). In addition, 51% (24/47) of the intervention group showed a clinically significant improvement in social anxiety (≥10-point reduction) compared to only 22% (10/45) of the control group (χ^2^_1_=6.3, *P*=.01). The percentage of participants reaching subclinical levels of social anxiety symptoms (SPIN score of ≤19) was not significantly different between the 2 groups (intervention: 9/47, 19%; waitlist control: 3/45, 7%; χ^2^_1_=2.1, *P*=.14).

At follow-up after 6 weeks, SPIN scores in the intervention group remained stable compared to the end of the intervention (mean reduction 0.05, SD 6.74), whereas the waitlist control group saw a mean reduction of 2.71 (SD 6.10) points (*t*_90_=1.97; FDR *P*=.05; Cohen *d*=0.43; Figure 5A). This might be because the waitlist control group had received access to the Alena app and 8% (4/50) were using it, whereas the intervention group no longer had access to the Alena app. The number of participants showing a significant reduction in SPIN scores was no longer significantly different between the 2 groups at this time point (intervention: 21/45, 47%; waitlist control: 15/44, 34%; χ^2^_1_=1.5, *P*=.47), and neither was the difference in reliable recovery between the groups (intervention: 8/45, 18%; waitlist control: 4/44, 9%; χ^2^_1_=0.8, *P*=.37).

These effects were broadly replicated in RCT 2. The intervention group showed a significantly larger reduction in SPIN scores (mean −12.89, SD 13.87) than that in the waitlist control group (mean −7.48, SD 12.24) by the end of the intervention period at 8 weeks (*t*_227_=−3.13; FDR *P*=.008; Cohen *d*=0.42; Figures 5C and 5D). A significantly larger proportion of the intervention group showed a clinically significant improvement in social anxiety compared to the control group (intervention: 63/105, 60%; control: 45/124, 36.3%; χ^2^_1_=12.9, *P*=.002), and a larger group of participants in the intervention group reached subclinical levels of social anxiety symptoms by the end of the 8-week intervention (intervention: 23/105, 21.9%; control: 13/124, 10.5%; χ^2^_1_=4.8, *P*=.006).

The effects in RCT 2 persisted at the week 12 follow-up even though participants in neither group had access to the Alena app during this time. Indeed, participants in the intervention group continued to show a reduction in SPIN scores compared to the end of the intervention period (intervention: mean −2.39, SD 6.15; control: mean −0.29, SD 6.41; *t*_227_=−2.48; FDR *P*=.03; Cohen *d*=0.33; Figure 5C). A larger proportion of participants assigned to the intervention group showed a clinically significant improvement in social anxiety (≥10-point reduction in SPIN scores; intervention: 65/104, 62.5%; control: 45/124, 36.3%; χ^2^_1_=17.0, *P*<.001), and they were 2.7 times more likely to have recovered (28/104, 26.9%) than waitlist control participants (14/121, 11.6%; χ^2^_1_=7.7, *P*=.006).

Finally, a linear mixed-effects regression analysis on SPIN scores over the intervention period (including baseline) modulated by group (intervention vs waitlist control) and controlling for age, sex (RCT 2 only), and the plateau effect of SPIN scores over time (week^2^) revealed a highly significant main effect of week (RCT 1: mean β −3.149, SD .398, *t*_392.719_=−7.922, and *P*<.001; RCT 2: mean β −3.388, SD .249, *t*_1855.835_=−13.589, and *P*<.001) and a group × week interaction in both RCTs (RCT 1: mean β 1.691, SD .566, *t*_391.935_=2.99, and *P*=.003; RCT 2: mean β 1.588, SD .340, *t*_1846.697_=4.669, and *P*<.001), suggesting that SPIN scores declined in both groups, but this decline was significantly steeper in the intervention group (see Table S1 in Multimedia Appendix 1 for details).

Overall, these results suggest that having access to the Alena app significantly reduced social anxiety symptoms beyond the decrease observed in the waitlist control group. Furthermore, both RCTs showed that this improvement persisted over time, suggesting a lasting impact of the Alena app.

### Daily Functioning

The impact of Alena on daily functioning was measured using the WSAS total scores. Participants in both groups experienced considerable functional impairment at baseline as measured using the WSAS. In RCT 1, the intervention group had a median WSAS score of 18.5 (IQR 12.00-25.00), whereas the waitlist group had a median score of 21.0 (IQR 15.25-24.00; *t*_100_=–0.59; *P*=.55). In RCT 2, the intervention group had a median WSAS score of 18.5 (IQR 14.00-24.25), whereas the waitlist group had a median score of 20.0 (IQR 12.00-24.00; *t*_247_=–0.06; *P*=.95).

**Figure 6:**
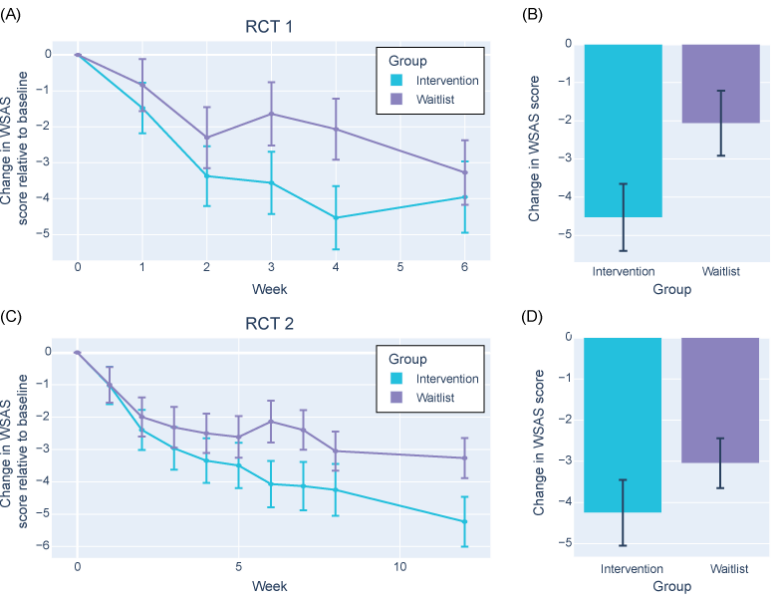
Improvement in daily functioning over time. (A) Mean change in Work and Social Adjustment Scale (WSAS) score across participants in either the intervention (blue) or the waitlist control (purple) group each week relative to the baseline assessment at week 0 in randomized controlled trial (RCT) 1. (B) Total change in WSAS score from week 0 to the final week of the intervention or waitlist period in RCT 1. (C) Mean change in WSAS score across participants in either the intervention (blue) or the waitlist control (purple) group each week relative to the baseline assessment at week 0 in RCT 2. (D) Total change in WSAS score from week 0 to the final week of the intervention or waitlist period in RCT 2. Error bars represent the SEM.

In RCT 1, by the end of the intervention, the intervention group showed a greater average reduction in WSAS scores (−4.53, SD 6.02) than the control group (−2.07, SD 5.71), although the difference was not statistically significant after adjusting for multiple comparisons (*t*_90_=−2.01; FDR *P*=.07; Cohen *d*=0.42; Figures 6A and B). At the 2-week follow-up, the control group experienced a slight improvement (mean −1.24, SD 2.77), whereas the intervention group’s scores slightly worsened (mean 1.05, SD 5.06), reaching statistical significance (*t*_82_=2.57; FDR *P*=.048; Cohen *d*=0.56).

In RCT 2, throughout the 8-week intervention period, both groups demonstrated improvements in WSAS scores, with the intervention group seeing a slightly larger average reduction (−4.25, SD 8.19) than the control group (−3.05, SD 6.74), although the difference was not significant (*t*_227_=−1.22; FDR *P*=.23; Cohen *d*=0.16; Figures 6C and D). At the week 12 follow-up, changes in WSAS scores were minimal and not significantly different between the intervention (mean −1.04, SD 4.36) and waitlist control group (mean 0.08, SD 4.22; *t*_227_=−1.93; FDR *P*=.07; Cohen *d*=0.26)

In an exploratory analysis, a linear mixed-effects regression model analyzed the WSAS scores over time adjusting for baseline values, group, age, and sex (in RCT 2 only) along with a quadratic time effect (week^2^). The analysis revealed a significant main effect of time, indicating that WSAS scores generally declined over the study period (RCT 1: mean β −1.572, SD .237, *t*_391.925_=−6.629, and *P*<.001; RCT 2: mean β −1.466, SD .152, *t*_1848.750_=−9.636, and *P*<.001). The group by time interaction was also significant, suggesting that the rate of decline in WSAS scores was steeper in the intervention group than in the control group (RCT 1: mean β .896, SD .337, *t*_391.329_=2.657, and *P*=.008; RCT 2: mean β .756, SD .207, *t*_1841.976_=3.646, and *P*<.001; see Table S2 in Multimedia Appendix 1 for details).

The data indicate that the Alena app had a positive, though variable, impact on reducing functional impairments associated with social anxiety as measured using the WSAS. The intervention group generally showed a greater improvement in daily functioning across both RCTs, particularly notable given the significant interaction effects in the mixed-effects models.

## Discussion

### Principal Findings

#### Overview

The findings of our 2 RCTs provide evidence for the efficacy, safety, and acceptability of a web-only, modularized iCBT program for social anxiety. In RCT 1, conducted in young women (aged 18-35 years), we found that the intervention was well tolerated, with no significant differences in adverse event frequency between the groups, high engagement levels, and a statistically significant reduction in social anxiety symptoms compared to the waitlist control. These results were replicated in RCT 2, which tested a broader sample of men and women aged 18 to 75 years. Participants in the intervention group again demonstrated greater symptom reduction than the control group, with similar effect sizes across the studies. Importantly, high retention and adherence rates suggest that the intervention was engaging and acceptable across demographics. Together, these findings indicate that a fully digital, modularized CBT approach can effectively reduce social anxiety symptoms in a web-based, self-guided format.

#### Safety

There were no indications that the web-based delivery of the interventions was unsafe. There was no increase in the severity or frequency of adverse events in the intervention group compared to the waitlist control group. The treatment included steps such as exposure, which is crucial for therapeutic benefit but necessarily induces discomfort and could in principle be unsafe. The interventions made this very explicit and provided instructions on how to engage in exposure safely. Hence, exposure can be delivered safely on the web without therapist involvement. This finding is also consistent with a recent meta-analysis that found that iCBT is generally safer than control conditions [38].

This study incorporated robust adverse event monitoring to digitally assess participant safety. By using weekly surveys to monitor and classify adverse events supported by blinded clinical review, the findings highlight a scalable model for adverse event tracking in digital mental health interventions. These methods address a critical gap in digital health research in which safety monitoring often lacks standardization or transparency. Our results demonstrate that digital therapies, when carefully designed and monitored, can be delivered safely without therapist involvement. This further validates the potential of stand-alone digital interventions as accessible and low-risk treatment options.

#### Acceptability

During the design of the app, detailed attention was paid to the user interface design, including an appealing visual design, easy-to-use interface, and bite-sized therapeutic content that was adapted to the participant population by providing relevant and normalizing examples. Overall, this led to high acceptability scores and positive user feedback. The high completion rate of the program (users completed 84%-91% of the material on the app) suggests that these features helped effectively engage users, encouraging consistent participation and adherence to the treatment protocol. As such, the results speak to the importance of careful user interface design for web-based interventions.

#### Efficacy

The app demonstrated robust efficacy across both a heterogeneous adult online sample and in a sample of women aged 18 to 35 years. The latter group is particularly important given the high prevalence of social anxiety in younger women. In both groups, the positive treatment effects persisted after the treatment period ended, indicating at least a short-term maintenance of the benefits.

Both RCT 1 and RCT 2 showed a significant symptom reduction in the intervention group compared to the waitlist control group, with similar effect sizes (Cohen *d*=0.47 in RCT 1 and Cohen *d*=0.42 in RCT 2). This suggests that the core CBT content shared across the 2 RCTs, including psychoeducational components supporting self-reflection, led to consistent improvements across different demographics despite differences in pacing and therapy access. High acceptability and adherence rates in both RCTs suggest that the app’s design effectively engaged participants, which could be crucial for maintaining efficacy across different samples and durations. The observed similar effect sizes in both RCTs suggest that adjustments in RCT 2—such as flexible pacing, extended intervention length, and additional non-CBT content included in the Community and Recharge tabs—had a negligible impact on the main effects of interest.

The efficacy of the Alena app either matched [39,40] or surpassed [9] that observed for previous digital interventions for social anxiety, although interventions including support from a human therapist can show enhanced effects [41]. In part, this probably reflects effective interface design driving engagement [42]. The efficacy observed in this study appears broadly comparable to in-person National Health Service (NHS) Talking Therapy outcomes. In 2022, improvement rates of 67.1% and recovery rates of 36.4% for social phobia disorder treated using CBT were reported by NHS Talking Therapies [43]. This is similar to the observed improvement rates of 62.5% (65/104) and recovery rates of 26.9% (28/104) in RCT 2. The NHS also reports an average reduction in WSAS scores of 5.8 points, which is similar to the average 5.2-point reduction (SD 7.86) we observed in RCT 2. Although it is not possible to compare clinical and self-selected online samples, these comparisons encourage the examination of the app’s efficacy in clinical settings given the substantial cost-effectiveness and scalable nature of a smartphone app.

### Limitations

There are several important limitations to consider. The first is the short follow-up time. During this time, control group participants in RCT 1 also received the active treatment. Given that SAD is often viewed as a chronic disorder, it is important to determine whether the treatment has sustained effects over a longer period.

Furthermore, social anxiety and functional impairments were assessed using questionnaire-based self-reports. These instruments are convenient and scalable, but they can be influenced by various biases, such as social desirability and recall biases or participants’ subjective interpretations of the questions. This may impact the accuracy of symptom severity and functional impairment assessments. In future studies, assessments of symptom severity by trained clinicians using structured clinical interviews could enhance the reliability and objectivity of symptom assessments. Clinical interviews are especially important for confirming participants’ diagnoses and ensuring that patients meet the criteria for SAD. Complementing self-report data with objective assessments would lead to clinically validated insights into participants’ symptoms and functional changes over time and might provide directions for further improving the treatment.

Another limitation concerns the sample, which was limited to active users of the recruitment website Prolific. Future studies should aim to include larger, more diverse samples. In particular, it is not possible to generalize the findings obtained from a self-selected online panel of volunteers to a clinical sample. As such, the applicability of our results to real-world clinical populations will need to be assessed.

In addition, we used a waitlist control as opposed to an active control condition as the comparison group in our study. Active controls, such as psychological placebos, often result in smaller effect sizes compared to passive controls [44,45]. This can be because participants in the waitlist control group are aware of their status, which can lead to expectancy effects and other nonspecific factors influencing the outcomes. However, our primary goal was to establish the initial safety, acceptability, and efficacy of the Alena app. A waitlist control allows for a clear comparison of the treatment effect without the confounding influence of another active intervention. Future studies should address this limitation by including active control groups to further validate the efficacy of the Alena app and mitigate potential biases. A direct comparison of digital interventions such as Alena with their face-to-face therapy counterparts would provide valuable insights into the relative strengths and limitations of digital therapy, helping refine these tools and better integrate them into mainstream mental health care. This direct comparison would also aid in identifying specific patient profiles that may benefit more from digital or traditional therapy modalities.

Although the therapy content was modularized, these studies delivered the interventions in a standardized fashion, with modules released in a fixed order.

While study app access was controlled and no unauthorized access was detected, it is possible that participants in the waitlist group accessed the publicly available version of the Alena app or used other mental health apps during the study period. This was not actively monitored and represents a potential limitation. However, any unreported app access or increased use of other apps by the waitlist group would likely have reduced the observed differences between the groups. This suggests that the reported effects of the intervention are conservative estimates.

Baseline data showed no significant differences in past therapy use or waitlist status between the intervention and waitlist control groups (Table 1), reducing the likelihood of confounding due to previous treatment history. However, a small proportion of participants in RCT 2 (12/124, 9.7% of the participants in the intervention group and 17/124, 13.7% of the participants in the control group) reported being on a waitlist for therapy at the start of the study (the frequency of participants reporting being on a waitlist for therapy was statistically equivalent between our 2 study arms), which could indicate potential access to therapeutic support during the study period. In addition, while no participants were actively undergoing therapy at baseline, the study did not monitor whether any participants initiated therapy during the trial. This represents a limitation as differences in access to parallel treatments between the groups could have influenced the observed effects. Future studies should include regular monitoring of concurrent treatments to better isolate the effects of digital interventions.

Finally, we cannot rule out that the efficacy of RCT 2 was due to engagement with the Recharge and Community content that participants in the intervention group had access to throughout the duration of the trial. Interaction with the non-CBT app features was not monitored in this study. However, participants in RCT 1 did not have access to this content, and effect sizes were comparable across the 2 trials. This suggests that the effects in RCT 2 can at least not be solely attributed to the additional content but likely resulted from the therapeutic interventions.

### Conclusions

In this study, iCBT retained efficacy despite the modularized format and the absence of a therapist intervention. This opens new doors for treating SAD by encouraging users to focus on specific cognitive and behavioral processes most relevant to their individual needs. The ability to tailor treatment plans based on individual profiles may ultimately help address the heterogeneity in symptom presentation and treatment response among individuals with SAD [27].

In the future, Alena could also be personalized to the needs of other psychological phenotypes beyond social anxiety that might benefit from similar tools. For example, individuals with generalized anxiety disorder might similarly find value in exposure therapy. By addressing the unique characteristics of various psychological phenotypes, Alena could increase user engagement, improve outcomes, and broaden its appeal across different mental health conditions. Furthermore, this level of customization could facilitate greater adoption in clinical settings, where practitioners often seek tools that complement personalized treatment plans. In this way, Alena has the potential to contribute meaningfully not only to individual well-being but also to the evolution of precision mental health care.

At the moment, the app holds promise as an intervention particularly for individuals with mild to moderate symptoms or those who face barriers to traditional treatment. Thus, as an accessible, low-intensity option, the app could serve as an initial step in care, reducing the burden on health care systems while offering early interventions to prevent symptom escalation and reduce the need for more intensive treatments. Future research should additionally explore the potential for integrating the app into existing primary mental health treatments. For example, the app could be used as an adjunct to therapy sessions, with app-generated insights or progress reports informing therapeutic strategies. In addition, it could facilitate symptom monitoring between sessions, providing clinicians with real-time data to support more personalized and timely interventions.

In conclusion, the Alena app exemplifies the substantial potential of digital therapy for SAD, adapting a gold standard model of CBT into a format that is safe, acceptable, effective, and highly scalable. These findings pave the way for the development of accessible and tailored treatments for individuals with SAD and other mental health conditions. By advancing our understanding of how to implement modularized CBT in a digital format effectively, we move closer to achieving more personalized and effective mental health care for all.

## Data Availability

The datasets generated or analyzed during this study are available in a GitHub repository [46].

## Appendix

Multimedia Appendix 1 Supplementary information containing a detailed intervention program outline, regression results, and adverse event reports.

Multimedia Appendix 2 CONSORT-eHEALTH checklist (V 1.6.1).

## Abbreviations

BF01: Bayes factor for the null hypothesis
CBT: cognitive behavioral therapy
CBT-SA: cognitive behavioral therapy for social anxiety
FDR: false discovery rate
iCBT: internet-delivered cognitive behavioral therapy
NHS: National Health Service
RCT: randomized controlled trial
SAD: social anxiety disorder
SPIN: Social Phobia Inventory
WSAS: Work and Social Adjustment Scale
