# Supplementary material for: Safety and Efficacy of Modular Digital Psychotherapy for Social Anxiety: Randomized Controlled Trial
Source: J Med Internet Res. 2025 Apr 10;27:e64138. doi: 10.2196/64138 (PMC12022530; doi:10.2196/64138)
Supplement: Multimedia Appendix 1 [file jmir_v27i1e64138_app1.pdf]

Supplementary Material

Safety and Efficacy of Modular Digital Psychotherapy for Social Anxiety: Randomized Controlled Trial

Mona M. Garvert*[1], Jessica MacFadyen*[1], Stuart Clarke[1], Tayla McCloud[1], Sofie Meyer[1], Sandra Sobanska[1], Paul B. Sharp[1], Alex Long[1], Quentin Huys#[1], Mandana Ahmadi#[1]

Program outline

RCT #1

Module	Format	Title	User-Facing Description
Introduction	Interactive worksheet	What is social anxiety?	This chapter is an overview of social anxiety. You'll gain an insight into your own symptoms and how to recognise them.
	Interactive worksheet	How does Alena work?	Learn more about Alena's unique approach, which combines evidence-based treatment with cutting-edge neuroscience assessments.
Beliefs	Interactive worksheet	Map out your social anxiety	In this exercise you will learn how to use an evidence-based tool called the Social Anxiety Map.
	Interactive worksheet	Understand your thought patterns	In this exercise, you will learn about the three types of negative thoughts which are common for social anxiety - worries, beliefs and images.
Attention	Interactive worksheet	What is self-attention?	In this explainer, you will learn more about negative self-attention and the role it plays in keeping social anxiety going.
	Interactive worksheet	Explore a new perspective	In this exercise you will work with a real situation from your life and learn how to see it from more than one perspective.
	Audio	Learn to shift your attention	In this guided meditation you will learn how to shift your attention by focusing on different objects or sounds around you.
	Audio	Practice shifting attention	In this exercise, you will practise this new skill in a situation you might encounter in the real world - giving a speech.
Avoidance	Interactive worksheet	What are safety behaviors?	Here you will learn more about avoidance and safety behaviors, and the role they play in social anxiety.
	Interactive worksheet	Prepare for real-life practice	This exercise will help you plan an experiment to drop a safety behavior in a real-life social situation.
	Interactive worksheet	Reflect on real-life practice	This reflection exercise will help you learn from your experiment and identify the most helpful next steps for you, in a non-judgemental and supportive way.
Rumination	Interactive worksheet	What is rumination?	In this explainer, you will learn more about rumination, how it can spiral, and the role this plays in keeping social anxiety going.
	Interactive worksheet	Learn to stop negative thoughts	In this exercise you will learn an evidence-based technique to help you break out of a negative thought spiral.
	Interactive worksheet	Challenge your memory bias	In this exercise, you will learn a simple way to challenge any tendency you may have to remember social events in a negatively biased way.

RCT #2

Module	Format	Title	User-Facing Description
Introduction	Assessment	Check your social anxiety level	Get started with this assessment to measure your level of social anxiety. You will use it to track your progress over time.
	Audio	What if it worked?	In this audio explainer, you will be guided to visualize what it would be like if working on your social anxiety with Alena worked.
	Interactive worksheet	Are you ready?	In this audio explainer, you will be guided to visualize how it would be like if working on your social anxiety with Alena worked.
	Interactive worksheet	Map out your social anxiety	In this exercise you will learn how to use an evidence-based tool called the Social Anxiety Map.
	Audio	What is social anxiety?	This chapter is an overview of social anxiety. You'll gain an insight into your own symptoms and how to recognise them.
	Audio	How does Alena work?	Learn more about Alena's unique approach, which combines evidence-based treatment with cutting-edge neuroscience assessments.
	Interactive worksheet	Quiz - Test your knowledge	This quiz is designed to help you refresh your memory and consolidate the information you learned in this module.
Beliefs	Assessment	Assess your beliefs	Understand how your beliefs may be driving your social anxiety with this interactive assessment.
	Audio	What are beliefs?	In this audio explainer, you will learn more about beliefs and the role they play in keeping social anxiety going.
	Interactive worksheet	Balance a belief	Learn an evidence-based technique to challenge an unhelpful belief and create a more balanced one.
	Interactive worksheet	Explore a new perspective	In this exercise you will work with a real situation from your life and learn how to see it from more than one perspective.
	Audio	Applying it to your life	In this audio explainer you'll learn how to start setting up experiments to start changing your beliefs in real life.
	Interactive worksheet	Put it into practice	In this exercise, you can set up your real-life experiment to challenge your beliefs, log your progress or journal about how it's going.
Attention	Assessment	Assess your attention	Play this fishing game where your goal is to work with a partner to catch as many fish as possible.
	Audio	What is self-attention?	In this audio explainer, you will learn more about negative self-attention and the role it plays in keeping social anxiety going.
	Audio	Visual attention training	In this guided meditation you will learn how to shift your attention by focusing on different objects around you.
	Audio	Applying it to your life	This audio explainer will help you review what you have learned about self-attention and how to apply it to your life.
	Interactive worksheet	Put it into practice	In this exercise, you can set up your real-life experiment to train your attention muscles, log your progress or journal about how it's going. Use this exercise to prepare guided experiments in real life situations.
Avoidance	Assessment	Assess your avoidance	In this assessment, you will be working with a partner and using your communication skills to gain tokens.
	Audio	What is avoidance?	In this audio explainer, you will learn more about avoidance and safety behaviors, and the role they play in social anxiety.
	Interactive worksheet	Identify a safety behaviour	In this exercise, you will reflect on one of your safety behaviors, and how it might be holding you back and keeping your anxiety going.

	Interactive worksheet	Plan your experiment	This exercise will help you plan an experiment to drop a safety behavior in a real-life social situation.
	Interactive worksheet	Reflect on your experiment	This reflection exercise will help you learn from your experiment and identify the most helpful next steps for you, in a non-judgemental and supportive way.
	Audio	Applying it to your life	This audio explainer will help you celebrate your progress, review what you have learned about avoidance, and learn how to continue expanding your "stretch zone".
	Interactive worksheet	Putting it into practice	In this exercise, you can set up experiments to drop your safety behaviors, log your progress or journal about how it's going.
Rumination	Assessment	Assess your rumination	You will play a code words game with others to uncover an impostor. Your goal is to guess the impostor and not get yourself caught!
	Audio	What is rumination?	In this audio explainer, you will learn more about rumination, how it can spiral, and the role this plays in keeping social anxiety going.
	Interactive worksheet	Learn to stop negative thoughts	In this exercise you will learn an evidence-based technique to help you break out of a negative thought spiral.
	Interactive worksheet	Challenge your memory bias	In this exercise, you will learn a simple way to challenge any tendency you may have to remember social events in a negatively biased way.
	Audio	Applying it to your life	This audio explainer will recap what you have learned about rumination and give you some helpful tips to for how to ruminate less.
	Interactive worksheet	Put it into practice	Now you've worked on the cognitive side of rumination, put it into practice by testing it out in a real life situation, reflecting about it in a journal entry, or logging your progress.

Supplementary Table 1. Linear mixed-effects regression analysis on the change in SPIN over the intervention period (including baseline but not including follow-up), modulated by group (intervention vs waitlist) and controlling for age, sex (RCT #2 only), and the plateau effect of SPIN over time (week^2). Significant effects are highlighted in yellow.

Formula: SPIN ~ group × week + week^2 + age + sex + (1|participant)

	Effect	Estimate (β)	Standard error	df	t value	p value	d
RCT #1	*Intercept*	*38.16*	*1.469*	110.986	*25.976*	*< 0.001 ****	
	Group	2.002	2.059	99.517	0.972	0.333	0.17
	Week	-3.149	0.398	392.719	-7.922	< 0.001 ***	0.27
	Week^2	0.406	0.336	391.396	1.207	0.228	0.03
	Age	1.408	1.03	99.318	1.367	0.175	0.12
	Group × Week	1.691	0.566	391.935	2.99	0.003**	0.15
RCT #2	*Intercept*	*36.044*	*1.226*	258.076	*29.399*	*< 0.001 ****	
	Group	2.885	1.689	244.928	1.708	0.89	0.24
	Week	-3.388	0.249	1855.835	-13.589	< 0.001***	0.28
	Week^2	1.092	0.154	1847.442	7.072	< 0.001***	0.09
	Age	0.019	0.677	246.764	0.028	0.978	0
	Sex	-1.788	1.988	255.102	-0.899	0.369	0.15
	Group × Week	1.588	0.340	1846.697	4.669	< 0.001***	0.13
	Group × Sex	-2.089	2.787	247.923	-0.750	0.454	0.17
	Week x Sex	-1.159	0.418	1869.403	-2.770	0.006**	0.10
	Group × Week × Sex	0.834	0.570	1854.456	1.462	0.144	0.07

d is an estimate of effect size, where d=varrand . Variance Inflation Factor (VIF) < 2.5.

Supplementary Table 2. Linear mixed-effects regression analysis on the change in WSAS over the intervention period (including baseline but not including follow-up), modulated by group (intervention vs waitlist) and controlling for age, sex (RCT #2 only), and the plateau effect of SPIN over time (week^2). Significant effects are highlighted in yellow.

Formula: WSAS ~ group × week + week^2 + age + sex + (1|participant)

RCT	Effect	Estimate (β)	Standard error	df	t value	p value	d
RCT #1	*Intercept*	*15.653*	*1.064*	106.993	*14.717*	*< 0.001****	
	Group	2.093	1.504	99.333	1.391	0.167	0.26
	Week	-1.572	0.237	391.925	-6.629	< 0.001***	0.19
	Week^2	0.418	0.2	390.944	2.088	0.037*	0.05
	Age	-0.014	0.752	99.184	-0.018	0.985	0
	Group × Week	0.896	0.337	391.329	2.657	0.008**	0.11
RCT #2	*Intercept*	*15.302*	*0.928*	253.128	*16.495*	*< 0.001****	
	Group	1.964	1.284	244.152	1.529	0.127	0.22
	Week	-1.466	.152	1848.750	-9.636	< 0.001***	0.17
	Week^2	0.443	0.094	1842.710	4.707	< 0.001***	0.05
	Age	0.497	0.514	245.486	0.966	0.335	0.06
	Sex	1.116	1.505	251.735	0.742	0.459	0.13
	Group × Sex	-2.769	2.116	246.367	-1.309	0.192	0.32
	Group × Week	0.756	0.207	1841.976	3.646	< 0.001***	0.09
	Week x Sex	-2.769	2.116	246.367	-1.309	0.192	0.32
	Group × Week × Sex	-0.450	0.348	1847.768	-1.293	0.196	0.05

d is an estimate of effect size, where d=varrand . Variance Inflation Factor (VIF) < 2.5.

Supplementary Information S3. Adverse health events reported by both groups

RCT #1

Intervention

Week 1: Just had a chest infection following Covid. Follow this with GP who prescribed antibiotics.
Week 2: Again chest infections being managed by antibiotics
Week 2: I got covid for the first time and I was hospitalised because of it. I was exhausted and in pain all week.
Week 2: Increased anxiety from not being able to complete the task - and then guilt for not being able to.
Week 2: It was hard with the speeches because I've had a horrible couple weeks and my anxiety has been all over the place so I didn't feel like I could do it at the point. But I maybe could when I wasn't feeling like that
Week 2: Brought some issues to the fore front for me
Week 3: Forcing me to face my emotions
Week 3: Anxious throwing up
Week 4: The speech task made me so anxious

Waitlist

Week 1: I have been feeling worse than normal. I have been in a few social situations and I always feel like the odd one out.
Week 1: Not being interested in anything
Week 1: Doing physical tasks around the house that have caused slight damage to muscles and joints.
Week 2: I'm having severe stomach issues, symptoms of early menopause also, feeling more down and low in myself than usual
Week 2: Having to go up the hospital with my daughter. The place drains me mentally and takes me awhile to get back to my normal self, didn't help that we were there for hours
Week 2: Feeling numb
Week 3: Concussion
Week 3: Unable to sleep due to anxiety about new job
Week 3: Breathing problems
Week 3: Discussion about ADHD or Bipolar diagnosis
Week 4: Concussion recovery
Week 4: Anxiety and sleep disruption caused by stress about new job
Week 4: Daughter fell down the stairs had to go hospital back up there again this Week due to her elbow not long coming out of plaster. Stressed and full of anxiety as she can't move her arm and I'm freaking out
Week 4: Really wanted to hurt myself. Urge

RCT #2

Intervention

Week 1: I think it just depends if you are a person who would rather talk to another person or if you are happy keeping it to an app
Week 1: Just feelings of sadness as I'm reflecting on things in my life
Week 1: I just found it difficult to believe some of the facts which resulted from the exercises. I thought it was too positive and not quite realistic
Week 1: The increased focusing on my feelings and the causes of them has been unsettling. Of course, this is not due to any fault with the app. I realise from experiences in therapy that this is normal.
Week 1: This is only very mild so not sure but I felt a little bit that I was constantly being told there was something wrong with me rather than feeling it was how I was and I felt this quite off-putting as I ended up especially at the start of the week feeling worse about myself than when I started.
Week 1: I have had a bad week so not sure if using the app has contributed towards this

Week 1: I don't think there were negative effects, but I noticed increase in negative thoughts, possibly because the excercise made me think.
Week 1: Not negative, but it has been uncomfortable thinking on issues and beliefs that I have. But I see it as a necessary part of the CBT.
Week 1: I found I asked myself some very fundamental questions which went to my core. I unexpectedly found myself very deeply upset as a result – distraught, even. It put me off using the app for a few days. I understand that this was probably a good thing but it might not be the best for someone with a depressive disposition. It was also partly my own fault, as I may have got too precise in my self analysis and I wasn't ready to confront the realities I found myself facing.
Week 2: Just the usual slight stress of focusing on my feelings of anxiety.
Week 2: I found myself rethinking my Traumatic period .
Week 2: Some of the exercises made me feel uncomfortable especially the guided meditation ones
Week 3: I wouldn't say it's been terrible but the fact it's making me reflect sometimes makes me feel like I blame myself for some of my thoughts as they might be unfounded
Week 3: I just get a little emotional when writing down how I feel and for what reason .it's quite repetitive
Week 3: I think I set myself too confrontational of challenges to meet. I've been avoiding using the app as a result.
Week 4: My physical tics are worse
Week 4: Had a downturn in mental health
Week 4: Hearing negative news
Week 4: Anxiety flair up, dermatology issues
Week 4: Increased tachycardia and chest pain (unrelated worsening of pre-existing Covid-related condition, probably)
Week 5: Some kind of virus
Week 5: Abnormal ecg
Week 6: I was very worried
Week 7: Im off work for depression and anxiety and this weeks been tough
Week 7: High blood pressure
Week 8: Feeling very stressed and vulnerable
Week 8: Just been really lethargic and not wanting to do anything
Week 8: I feel disappointed in myself as I have got stuck. It's as likely to be very particular circumstances this Week but I don't feel like I can push forward with challenging myself. I haven't had time to do the more reflective exercises this week which I would have liked to do and might have helped.
Week 8: I just felt. Little anxious when recalling
Week 8: My general mood was very low
Week 8: Just been really lethargic and not wanting to do anything

Waitlist
Week 4: I lost my job, I was dismissed on health grounds because I was no longer capable of doing my job after working for the company for 12 years.
Week 4: I have been experiencing an increasing level of stress due to a personal commitment that's coming up soon (exams). This isn't related to my participation in your study.
Week 4: Covid
Week 4: Very fatigued so feeling rather low in mood. No energy to do anything.
Week 4: I have been diagnosed with high blood pressure and put on medication immediately. This has left me scared and anxious for my health.
Week 4: Had bad news for a family member
Week 4: It is only physical discomfort because I am Covid positive .
Week 4: Chest infection
Week 5: I have been eating and drinking too much to counteract the loneliness and uselessness I feel. It's been a bank holiday and I've done nothing but mope!
Week 5: Anxiety and stress spiked, causing me to go on SSRIs
Week 5: Hallucinations, can't close my eyes without seeing unpleasant so really affecting my sleep
Week 5: Chest infection/ throat infection
Week 5: I've developed several unrelated symptoms like muscle twitching, dizziness and clumsiness. The issue is under investigation at the moment.

Week 6: I can't finish things. My house is full of 'stuff' crafts I decided to take up but didn't keep up with, clothes that don't fit me, bags of I don't know what everywhere. I eat and drink far too much. I buy more clothes and brick a brac that I can't afford!
Week 6: Heightened mental health issues due to change in circumstances
Week 6: Worsening of depression and low mood
Week 6: Leg pain
Week 6: Intense loneliness
Week 6: Very bad allergic reaction to caterpillars
Week 6: anxiety
Week 7: I've turned to alcohol to try to shut out everything
Week 7: Assaulted at work resulting in a fractured nose
Week 7: Panic attacks
Week 7: I had a hospital appointment about my eye which is struggling with very poor vision. The doctor diagnosed a cataract so I am on the waiting list for surgery. I feel better for knowing at least
Week 8: I have had to go for blood tests to do with my epilepsy and I'm concerned about the results that I don't know at the present
Week 8: Breathing difficulty
Week 8: Anxiety
Week 8: I have not experienced any negative health effects arising out of my participation in this study. However, I have been experiencing poor treatment while at work and ultimately resigned with immediate effect yesterday because the impact it was having on my mental health wasn't worth the (mediocre) pay.
Week 8: I had a terribly acute sense of being overwhelmed and powerless.

Summary

Participants on the intervention arm experienced a mix of physical and psychological challenges. Early weeks were dominated by health complications such as chest infections and the lingering effects of COVID-19, which in one case required hospitalization. These physical setbacks were compounded by psychological symptoms, with one participant reporting significant anxiety leading to physical symptoms like vomiting.

Participants in the waitlist arm reported a broader range of adverse events, including emotional and physical distress. Common themes included feelings of disinterest, numbness, and worsening mood, which are often associated with untreated mental health conditions. Physical injuries from household tasks and concussion further complicated their experiences, as did external stressors such as caregiving challenges and hospitalization visits for family members. Notably, severe emotional distress was documented, with one participant describing an urge to self-harm, underscoring the need for ongoing mental health support, even for those awaiting intervention.

In the second RCT, adverse events in the intervention arm appeared more psychological in nature, including reports of worsened physical tics, stress, lethargy, and feelings of vulnerability. Participants also expressed frustration with themselves, citing challenges in adhering to reflective exercises and feelings of stagnation.

For participants in the waitlist arm, adverse events spanned mental and physical health domains. Several individuals experienced infections (chest and throat) alongside heightened mental health symptoms such as anxiety, stress, hallucinations, and depression. One participant reported turning to alcohol as a coping mechanism, illustrating the potential risks of leaving individuals without active intervention. The overlap of health concerns, such as epilepsy-related blood tests and breathing difficulties, further compounded these challenges.
