# Supplementary material for: Safety and Efficacy of Modular Digital Psychotherapy for Social Anxiety: Randomized Controlled Trial
Source: J Med Internet Res. 2025 Apr 10;27:e64138. doi: 10.2196/64138 (PMC12022530; doi:10.2196/64138)
Supplement: Multimedia Appendix 2 [file jmir_v27i1e64138_app2.pdf]

CONSORT-EHEALTH (V 1.6.1) - Submission/Publication Form

The CONSORT-EHEALTH checklist is intended for authors of randomized trials evaluating web-based and Internet-based applications/interventions, including mobile interventions, electronic games (incl multiplayer games), social media, certain telehealth applications, and other interactive and/or networked electronic applications. Some of the items (e.g. all subitems under item 5 - description of the intervention) may also be applicable for other study designs.

The goal of the CONSORT EHEALTH checklist and guideline is to be
a) a guide for reporting for authors of RCTs,
b) to form a basis for appraisal of an ehealth trial (in terms of validity)

CONSORT-EHEALTH items/subitems are MANDATORY reporting items for studies published in the Journal of Medical Internet Research and other journals / scientific societies endorsing the checklist.

Items numbered 1., 2., 3., 4a., 4b etc are original CONSORT or CONSORT-NPT (non-pharmacologic treatment) items.
Items with Roman numerals (i., ii, iii, iv etc.) are CONSORT-EHEALTH extensions/clarifications.

As the CONSORT-EHEALTH checklist is still considered in a formative stage, we would ask that you also RATE ON A SCALE OF 1-5 how important/useful you feel each item is FOR THE PURPOSE OF THE CHECKLIST and reporting guideline (optional).

Mandatory reporting items are marked with a red *.
In the textboxes, either copy & paste the relevant sections from your manuscript into this form - please include any quotes from your manuscript in QUOTATION MARKS,
or answer directly by providing additional information not in the manuscript, or elaborating on why the item was not relevant for this study.

YOUR ANSWERS WILL BE PUBLISHED AS A SUPPLEMENTARY FILE TO YOUR PUBLICATION IN JMIR AND ARE CONSIDERED PART OF YOUR PUBLICATION (IF ACCEPTED).
Please fill in these questions diligently. Information will not be copyedited, so please use proper spelling and grammar, use correct capitalization, and avoid abbreviations.

DO NOT FORGET TO SAVE AS PDF _AND_ CLICK THE SUBMIT BUTTON SO YOUR ANSWERS ARE IN OUR DATABASE !!!

Citation Suggestion (if you append the pdf as Appendix we suggest to cite this paper in the caption):
Eysenbach G, CONSORT-EHEALTH Group
[illegible]

Ihre Antwort ist zu lang. Kürzen Sie einige Sätze in Ihrer Antwort.

[illegible]

URL: http://www.jmir.org/2011/4/e126/
doi: 10.2196/jmir.1923
PMID: 22209829

mona.garvert@gmail.com Konto wechseln

Nicht freigegeben

Entwurf gespeichert

* Gibt eine erforderliche Frage an

Your name *

First Last

Mona Garvert

Primary Affiliation (short), City, Country *

University of Toronto, Toronto, Canada

Alena, London, UK

Your e-mail address *

abc@gmail.com

mona.garvert@gmail.com

Title of your manuscript *

Provide the (draft) title of your manuscript.

Safety and efficacy of a modular digital psychotherapy for social anxiety: A randomized controlled trial

Ihre Antwort ist zu lang. Kürzen Sie einige Sätze in Ihrer Antwort.

Name of your App/Software/Intervention *

If there is a short and a long/alternate name, write the short name first and add the long name in brackets.

Alena

Evaluated Version (if any)

e.g. "V1", "Release 2017-03-01", "Version 2.0.27913"

Meine Antwort

Language(s) *

What language is the intervention/app in? If multiple languages are available, separate by comma (e.g. "English, French")

English

URL of your Intervention Website or App

e.g. a direct link to the mobile app on app in appstore (itunes, Google Play), or URL of the website. If the intervention is a DVD or hardware, you can also link to an Amazon page.

Meine Antwort

URL of an image/screenshot (optional)

Meine Antwort

Ihre Antwort ist zu lang. Kürzen Sie einige Sätze in Ihrer Antwort.

Accessibility *

Can an enduser access the intervention presently?

- ( ) access is free and open
- ( ) access only for special usergroups, not open
- ( ) access is open to everyone, but requires payment/subscription/in-app purchases
- (•) app/intervention no longer accessible
- ( ) Sonstiges:

Primary Medical Indication/Disease/Condition *

e.g. "Stress", "Diabetes", or define the target group in brackets after the condition, e.g. "Autism (Parents of children with)", "Alzheimers (Informal Caregivers of)"

Social anxiety

Primary Outcomes measured in trial *

comma-separated list of primary outcomes reported in the trial

RCT 1: Serious adverse events, Satisfaction, he

Secondary/other outcomes

Are there any other outcomes the intervention is expected to affect?

RCT 1: Change in Social Phobia Inventory (SPIN), Change in Work and Social Adjustment Scale (WSAS), RCT 2: Serious adverse events

Ihre Antwort ist zu lang. Kürzen Sie einige Sätze in Ihrer Antwort.

Recommended "Dose" *

What do the instructions for users say on how often the app should be used?

- (selected) Approximately Daily
- Approximately Weekly
- Approximately Monthly
- Approximately Yearly
- "as needed"
- Sonstiges:

Approx. Percentage of Users (starters) still using the app as recommended after 3 months *

- (selected) unknown / not evaluated
- 0-10%
- 11-20%
- 21-30%
- 31-40%
- 41-50%
- 51-60%
- 61-70%
- 71%-80%
- 81-90%
- 91-100%
- Sonstiges:

Ihre Antwort ist zu lang. Kürzen Sie einige Sätze in Ihrer Antwort.

Overall, was the app/intervention effective? *

- (x) yes: all primary outcomes were significantly better in intervention group vs control
- ( ) partly: SOME primary outcomes were significantly better in intervention group vs control
- ( ) no statistically significant difference between control and intervention
- ( ) potentially harmful: control was significantly better than intervention in one or more outcomes
- ( ) inconclusive: more research is needed
- ( ) Sonstiges:

Article Preparation Status/Stage *

At which stage in your article preparation are you currently (at the time you fill in this form)

- ( ) not submitted yet - in early draft status
- ( ) not submitted yet - in late draft status, just before submission
- (x) submitted to a journal but not reviewed yet
- ( ) submitted to a journal and after receiving initial reviewer comments
- ( ) submitted to a journal and accepted, but not published yet
- ( ) published
- ( ) Sonstiges:

Ihre Antwort ist zu lang. Kürzen Sie einige Sätze in Ihrer Antwort.

Journal *

If you already know where you will submit this paper (or if it is already submitted), please provide the journal name (if it is not JMIR, provide the journal name under "other")

- ( ) not submitted yet / unclear where I will submit this
- (•) Journal of Medical Internet Research (JMIR)
- ( ) JMIR mHealth and UHealth
- ( ) JMIR Serious Games
- ( ) JMIR Mental Health
- ( ) JMIR Public Health
- ( ) JMIR Formative Research
- ( ) Other JMIR sister journal
- ( ) Sonstiges:

Is this a full powered effectiveness trial or a pilot/feasibility trial? *

- ( ) Pilot/feasibility
- (•) Fully powered

Manuscript tracking number *

If this is a JMIR submission, please provide the manuscript tracking number under "other" (The ms tracking number can be found in the submission acknowledgement email, or when you login as author in JMIR. If the paper is already published in JMIR, then the ms tracking number is the four-digit number at the end of the DOI, to be found at the bottom of each published article in JMIR)

- ( ) no ms number (yet) / not (yet) submitted to / published in JMIR
- (•) Sonstiges: ms#64138

Ihre Antwort ist zu lang. Kürzen Sie einige Sätze in Ihrer Antwort.

TITLE AND ABSTRACT

1a) TITLE: Identification as a randomized trial in the title

1a) Does your paper address CONSORT item 1a? *

I.e does the title contain the phrase "Randomized Controlled Trial"? (if not, explain the reason under "other")

- (●) yes
- ( ) Sonstiges:

1a-i) Identify the mode of delivery in the title

Identify the mode of delivery. Preferably use “web-based” and/or “mobile” and/or “electronic game” in the title. Avoid ambiguous terms like “online”, “virtual”, “interactive”. Use “Internet-based” only if Intervention includes non-web-based Internet components (e.g. email), use “computer-based” or “electronic” only if offline products are used. Use “virtual” only in the context of “virtual reality” (3-D worlds). Use “online” only in the context of “online support groups”. Complement or substitute product names with broader terms for the class of products (such as “mobile” or “smart phone” instead of “iphone”), especially if the application runs on different platforms.

	1	2	3	4	5	
subitem not at all important	( )	( )	( )	(●)	( )	essential

Auswahl löschen

Does your paper address subitem 1a-i? *

Copy and paste relevant sections from manuscript title (include quotes in quotation marks "like this" to indicate direct quotes from your manuscript), or elaborate on this item by providing additional information not in the ms, or briefly explain why the item is not applicable/relevant for your study

"Safety and efficacy of a modular digital psychotherapy for social anxiety: A randomized

Ihre Antwort ist zu lang. Kürzen Sie einige Sätze in Ihrer Antwort.

1a-ii) Non-web-based components or important co-interventions in title

Mention non-web-based components or important co-interventions in title, if any (e.g., “with telephone support”).

	1	2	3	4	5	
subitem not at all important	●	○	○	○	○	essential

Auswahl löschen

Does your paper address subitem 1a-ii?

Copy and paste relevant sections from manuscript title (include quotes in quotation marks "like this" to indicate direct quotes from your manuscript), or elaborate on this item by providing additional information not in the ms, or briefly explain why the item is not applicable/relevant for your study

Meine Antwort

1a-iii) Primary condition or target group in the title

Mention primary condition or target group in the title, if any (e.g., “for children with Type I Diabetes”) Example: A Web-based and Mobile Intervention with Telephone Support for Children with Type I Diabetes: Randomized Controlled Trial

	1	2	3	4	5	
subitem not at all important	○	○	○	○	●	essential

Auswahl löschen

Does your paper address subitem 1a-iii? *

Copy and paste relevant sections from manuscript title (include quotes in quotation marks "like this" to indicate direct quotes from your manuscript), or elaborate on this item by providing additional information not in the ms, or briefly explain why the item is not applicable/relevant for your study

[illegible]

Ihre Antwort ist zu lang. Kürzen Sie einige Sätze in Ihrer Antwort.

1b) ABSTRACT: Structured summary of trial design, methods, results, and conclusions

NPT extension: Description of experimental treatment, comparator, care providers, centers, and blinding status.

1b-i) Key features/functionalities/components of the intervention and comparator in the METHODS section of the ABSTRACT

Mention key features/functionalities/components of the intervention and comparator in the abstract. If possible, also mention theories and principles used for designing the site. Keep in mind the needs of systematic reviewers and indexers by including important synonyms. (Note: Only report in the abstract what the main paper is reporting. If this information is missing from the main body of text, consider adding it)

	1	2	3	4	5	
subitem not at all important	○	○	○	○	◉	essential

Auswahl löschen

Does your paper address subitem 1b-i? *

Copy and paste relevant sections from the manuscript abstract (include quotes in quotation marks "like this" to indicate direct quotes from your manuscript), or elaborate on this item by providing additional information not in the ms, or briefly explain why the item is not applicable/relevant for your study

"We developed a novel digital adaptation of CBT for social anxiety that is both modular and fully digital without therapist in the loop and implemented it in a smartphone app."

Ihre Antwort ist zu lang. Kürzen Sie einige Sätze in Ihrer Antwort.

1b-ii) Level of human involvement in the METHODS section of the ABSTRACT

Clarify the level of human involvement in the abstract, e.g., use phrases like “fully automated” vs. “therapist/nurse/care provider/physician-assisted” (mention number and expertise of providers involved, if any). (Note: Only report in the abstract what the main paper is reporting. If this information is missing from the main body of text, consider adding it)

	1	2	3	4	5	
subitem not at all important	○	○	○	○	◉	essential

Auswahl löschen

Does your paper address subitem 1b-ii?

Copy and paste relevant sections from the manuscript abstract (include quotes in quotation marks "like this" to indicate direct quotes from your manuscript), or elaborate on this item by providing additional information not in the ms, or briefly explain why the item is not applicable/relevant for your study

"We developed a novel digital adaptation of CBT for social anxiety that is both modular and fully digital without therapist in the loop"

Ihre Antwort ist zu lang. Kürzen Sie einige Sätze in Ihrer Antwort.

1b-iii) Open vs. closed, web-based (self-assessment) vs. face-to-face assessments in the METHODS section of the ABSTRACT

Mention how participants were recruited (online vs. offline), e.g., from an open access website or from a clinic or a closed online user group (closed usergroup trial), and clarify if this was a purely web-based trial, or there were face-to-face components (as part of the intervention or for assessment). Clearly say if outcomes were self-assessed through questionnaires (as common in web-based trials). Note: In traditional offline trials, an open trial (open-label trial) is a type of clinical trial in which both the researchers and participants know which treatment is being administered. To avoid confusion, use “blinded” or “unblinded” to indicated the level of blinding instead of “open”, as “open” in web-based trials usually refers to “open access” (i.e. participants can self-enrol). (Note: Only report in the abstract what the main paper is reporting. If this information is missing from the main body of text, consider adding it)

	1	2	3	4	5	
subitem not at all important	○	○	○	○	◉	essential

Auswahl löschen

Does your paper address subitem 1b-iii?

Copy and paste relevant sections from the manuscript abstract (include quotes in quotation marks "like this" to indicate direct quotes from your manuscript), or elaborate on this item by providing additional information not in the ms, or briefly explain why the item is not applicable/relevant for your study

The study used two web-based, unblinded, randomized controlled trials (RCTs) without any face-to-face components. All assessments and interventions were conducted online through a smartphone app.
Participants were recruited online using Prolific, an online recruitment platform. This open access approach allowed self-enrollment by participants who met the study's eligibility criteria. Outcomes were self-assessed through online questionnaires. Participants completed these questionnaires, such as the Social Phobia Inventory (SPIN) and the Work and Social Adjustment Scale (WSAS), every week during the intervention or waitlist period. Both trials were unblinded, both the participants and the researchers knew which treatment was being administered.

Ihre Antwort ist zu lang. Kürzen Sie einige Sätze in Ihrer Antwort.

1b-iv) RESULTS section in abstract must contain use data

Report number of participants enrolled/assessed in each group, the use/uptake of the intervention (e.g., attrition/adherence metrics, use over time, number of logins etc.), in addition to primary/secondary outcomes. (Note: Only report in the abstract what the main paper is reporting. If this information is missing from the main body of text, consider adding it)

	1	2	3	4	5	
subitem not at all important	○	○	○	○	●	essential

Auswahl löschen

Does your paper address subitem 1b-iv?

Copy and paste relevant sections from the manuscript abstract (include quotes in quotation marks "like this" to indicate direct quotes from your manuscript), or elaborate on this item by providing additional information not in the ms, or briefly explain why the item is not applicable/relevant for your study

The following information is contained in the abstract: "Primary outcomes were safety and efficacy over 6 weeks in 102 women aged 18-35 (RCT #1) and symptom reduction (Social Phobia Inventory total scores) after 8 weeks in 267 men and women aged 18-75 (RCT #2)."

Information about attrition/adherence is reported in the manuscript:
"Even though participants were not incentivised to adhere to the therapy program (they were only compensated for the time required to complete the weekly surveys), participants in RCT #1 showed a median completion rate of 90.91% (mean = 76.92%, SD = 29.17%, Figure 6). For RCT #2, which featured a longer treatment program, the median completion rate was 84.85% (mean = 73.83%, SD = 27.89%)."

Ihre Antwort ist zu lang. Kürzen Sie einige Sätze in Ihrer Antwort.

1b-v) CONCLUSIONS/DISCUSSION in abstract for negative trials

Conclusions/Discussions in abstract for negative trials: Discuss the primary outcome - if the trial is negative (primary outcome not changed), and the intervention was not used, discuss whether negative results are attributable to lack of uptake and discuss reasons. (Note: Only report in the abstract what the main paper is reporting. If this information is missing from the main body of text, consider adding it)

	1	2	3	4	5	
subitem not at all important	(x)	( )	( )	( )	( )	essential

Auswahl löschen

Does your paper address subitem 1b-v?

Copy and paste relevant sections from the manuscript abstract (include quotes in quotation marks "like this" to indicate direct quotes from your manuscript), or elaborate on this item by providing additional information not in the ms, or briefly explain why the item is not applicable/relevant for your study

Primary outcome is not negative.

INTRODUCTION

2a) In INTRODUCTION: Scientific background and explanation of rationale

Ihre Antwort ist zu lang. Kürzen Sie einige Sätze in Ihrer Antwort.

2a-i) Problem and the type of system/solution

Describe the problem and the type of system/solution that is object of the study: intended as stand-alone intervention vs. incorporated in broader health care program? Intended for a particular patient population? Goals of the intervention, e.g., being more cost-effective to other interventions, replace or complement other solutions? (Note: Details about the intervention are provided in “Methods” under 5)

	1	2	3	4	5	
subitem not at all important	○	○	○	○	◉	essential

Auswahl löschen

Does your paper address subitem 2a-i? *

Copy and paste relevant sections from the manuscript (include quotes in quotation marks "like this" to indicate direct quotes from your manuscript), or elaborate on this item by providing additional information not in the ms, or briefly explain why the item is not applicable/relevant for your study

Problem: "Social anxiety disorder (SAD) is a prevalent and debilitating mental health challenge, affecting a substantial portion of the global population at some point in their lives. Characterized by a persistent fear of social situations, SAD can severely limit a person’s ability to engage in everyday activities, from forming personal relationships to navigating work and educational settings. In the long term, SAD can lead to profound social isolation, missed opportunities, and comorbid conditions such as depression, generalized anxiety disorder, and substance abuse" .

Type of System/Solution: "We developed an online-only, modular, iCBT program based on Clark and Wells’ therapy. In it, separate modules target each of the core cognitive components in the standard treatment, including negative beliefs, self-focused attention, rumination, and avoidance behaviors" .

The intervention targets individuals with social anxiety disorder.

The goals of the intervention are:
To provide a cost-effective alternative to traditional therapy, addressing the "limitations in accessibility and effectiveness" of traditional CBT-SA.
To offer a "modular and fully digital" therapy option that allows individuals to engage in treatment at their own pace and in their own environment, thus overcoming barriers related to therapist availability and patient reluctance to seek help .
By providing a modular approach, the intervention aims to "tailor treatments to individual cognitive profiles" to improve efficacy. The modular system allows for the personalization of therapy, potentially leading to more significant and faster improvements in symptoms .

Ihre Antwort ist zu lang. Kürzen Sie einige Sätze in Ihrer Antwort.

2a-ii) Scientific background, rationale: What is known about the (type of) system

Scientific background, rationale: What is known about the (type of) system that is the object of the study (be sure to discuss the use of similar systems for other conditions/diagnoses, if appropiate), motivation for the study, i.e. what are the reasons for and what is the context for this specific study, from which stakeholder viewpoint is the study performed, potential impact of findings [2]. Briefly justify the choice of the comparator.

	1	2	3	4	5	
subitem not at all important	○	○	○	○	◉	essential

Auswahl löschen

Ihre Antwort ist zu lang. Kürzen Sie einige Sätze in Ihrer Antwort.

Does your paper address subitem 2a-ii? *

Copy and paste relevant sections from the manuscript (include quotes in quotation marks "like this" to indicate direct quotes from your manuscript), or elaborate on this item by providing additional information not in the ms, or briefly explain why the item is not applicable/relevant for your study

"Although by its very nature it often presents late, cognitive-behavioral therapy for social anxiety (CBT-SA) has been established as an effective treatment with moderate to large effects on social anxiety symptoms [5–7]. CBT-SA typically addresses the cognitive processes and behavioral patterns that sustain social anxiety, including negative self-perception in social interactions, self-directed attention, anticipatory and post-event processing of social situations and safety behaviors that, paradoxically, maintain anxiety because they prevent the disconfirmation of negative beliefs [8]. By challenging these patterns, CBT facilitates significant improvements in symptoms, enabling individuals to engage more freely in social situations.
However, traditional CBT-SA faces limitations in accessibility [9] and effectiveness. A significant portion of those suffering from SAD never seek treatment, because interacting with a therapist, a cornerstone of traditional CBT, can be a phobic stimulus [10,11]. Moreover, in many places around the world the availability of trained therapists cannot meet the demand, leading to long wait times and further barriers to accessing care [12]. In response to these challenges, internet-delivered CBT (iCBT), has emerged as promising alternative to traditional therapy [7,13–18]. These programs offer individuals the opportunity to work through therapeutic exercises and techniques at their own pace and in the comfort of their own environment and provide discreet, affordable and immediate support to those in need [19]. iCBT can be supported by a therapist providing guidance remotely throughout the treatment process or be completely self-guided [20–22]. Recent meta-analyses showed that iCBT with and without therapist involvement can be effective in reducing symptoms of SAD [9,22–26].
CBT-SA also does not always work, and can be slow [27]. This may be because standard CBT involves a broad range of interventions aimed at various cognitive and behavioral processes, whereas individual patients may benefit predominantly from a specific subset of these interventions [28,29]. It may hence be possible to further improve treatment efficacy and speed by tailoring interventions to individual cognitive or behavioral profiles [30]. This requires breaking down CBT-SA into distinct, separable modules that target specific cognitive processes or mechanisms selectively [31].
Personalization of treatment may then be achieved by matching interventions to a person's cognitive profile."

2b) In INTRODUCTION: Specific objectives or hypotheses

Ihre Antwort ist zu lang. Kürzen Sie einige Sätze in Ihrer Antwort.

Does your paper address CONSORT subitem 2b? *

Copy and paste relevant sections from the manuscript (include quotes in quotation marks "like this" to indicate direct quotes from your manuscript), or elaborate on this item by providing additional information not in the ms, or briefly explain why the item is not applicable/relevant for your study

"To address accessibility and to work towards a modular targeted therapy, we developed an online-only, modular, iCBT program based on Clark and Wells' therapy [32]. In it, separate modules target each of the core cognitive components in the standard treatment, including negative beliefs, self-focused attention, rumination, and avoidance behaviors.
Here, we report the findings from two randomized controlled trials investigating the safety, acceptability, and efficacy of this iCBT program in the form of a smartphone app. Overall, the treatment remained safe, acceptable, and effective, significantly improving symptoms of social anxiety in two separate samples compared to a waitlist control group (RCT #1: N = 102, RCT #2: N = 267)."

METHODS

3a) Description of trial design (such as parallel, factorial) including allocation ratio

Does your paper address CONSORT subitem 3a? *

Copy and paste relevant sections from the manuscript (include quotes in quotation marks "like this" to indicate direct quotes from your manuscript), or elaborate on this item by providing additional information not in the ms, or briefly explain why the item is not applicable/relevant for your study

"We conducted two web-based, unblinded, randomized controlled trials (RCTs). RCT #1 was a six-week parallel-group randomized controlled trial with a four-week intervention and a two-week follow-up. RCT #2 was an eight-week trial with a four-week follow-up. In both trials, participants were randomized 1:1 to the active arm with access to the smartphone app, or the control arm without access to the smartphone app. In RCT #1, participants in the control arm were given access to the smartphone app at week 4, in RCT #2 at week 12."

3b) Important changes to methods after trial commencement (such as eligibility

Ihre Antwort ist zu lang. Kürzen Sie einige Sätze in Ihrer Antwort.

Does your paper address CONSORT subitem 3b? *

Copy and paste relevant sections from the manuscript (include quotes in quotation marks "like this" to indicate direct quotes from your manuscript), or elaborate on this item by providing additional information not in the ms, or briefly explain why the item is not applicable/relevant for your study

Yes. No methods were changed after trial commencement.

3b-i) Bug fixes, Downtimes, Content Changes

Bug fixes, Downtimes, Content Changes: ehealth systems are often dynamic systems. A description of changes to methods therefore also includes important changes made on the intervention or comparator during the trial (e.g., major bug fixes or changes in the functionality or content) (5-iii) and other "unexpected events" that may have influenced study design such as staff changes, system failures/downtimes, etc. [2].

	1	2	3	4	5	
subitem not at all important	(x)	( )	( )	( )	( )	essential

Auswahl löschen

Does your paper address subitem 3b-i?

Copy and paste relevant sections from the manuscript (include quotes in quotation marks "like this" to indicate direct quotes from your manuscript), or elaborate on this item by providing additional information not in the ms, or briefly explain why the item is not applicable/relevant for your study

The app was frozen before trial commencement. No bug fixes, downtimes or content changes occurred during the trial.

4a) Eligibility criteria for participants

Ihre Antwort ist zu lang. Kürzen Sie einige Sätze in Ihrer Antwort.

Does your paper address CONSORT subitem 4a? *

Copy and paste relevant sections from the manuscript (include quotes in quotation marks "like this" to indicate direct quotes from your manuscript), or elaborate on this item by providing additional information not in the ms, or briefly explain why the item is not applicable/relevant for your study

"Inclusion criteria were as follows:
1. Social Anxiety Symptom Severity: SPIN total score of 30 or higher, indicating a moderate to severe level of social anxiety.
2. Stability on Mental Health Medication: unchanged dose for 8 weeks or more.
3. Age: For RCT #1, participants had to be between 18 and 35 years old. For RCT #2, participants had to be between 18 and 75 years old.
4. Female [RCT #1 only]
5. Technology [RCT #1 only]: smartphone with iOS and internet access. [RCT #2 only]: smartphone with internet access and android or iOS operating system.

Exclusion criteria for both studies were as follows:
1. Alcohol Use: The Alcohol Use Disorders Identification Test for Consumption (AUDIT-C, [36] was used to assess the risk of alcohol dependence. Eligibility required participants to report less than a severe risk level (below eight points out of a possible 12).
2. Recreational Drug Usage: Participants were screened for recreational drug use with the following three questions: Have you used any recreational drugs in the last three months? In the last three months, have you had a strong desire or urge to use recreational drugs at least once a week or more often? In the last three months, has anyone expressed concern about your use of recreational drugs? Eligibility was limited to those reporting minimal to no use (below two points out of a possible 3).
3. Prior Use of Alena App: Participants who had previously used the Alena app were excluded."

4a-i) Computer / Internet literacy

Computer / Internet literacy is often an implicit “de facto” eligibility criterion - this should be explicitly clarified.

	1	2	3	4	5	
subitem not at all important	○	○	○	○	◉	essential

Auswahl löschen

Ihre Antwort ist zu lang. Kürzen Sie einige Sätze in Ihrer Antwort.

Does your paper address subitem 4a-i?

Copy and paste relevant sections from the manuscript (include quotes in quotation marks "like this" to indicate direct quotes from your manuscript), or elaborate on this item by providing additional information not in the ms, or briefly explain why the item is not applicable/relevant for your study

Participants needed to have a "smartphone with iOS and internet access" for RCT #1 and a "smartphone with internet access and android or iOS operating system" for RCT #2 . This requires a basic level of computer and internet literacy.

4a-ii) Open vs. closed, web-based vs. face-to-face assessments:

Open vs. closed, web-based vs. face-to-face assessments: Mention how participants were recruited (online vs. offline), e.g., from an open access website or from a clinic, and clarify if this was a purely web-based trial, or there were face-to-face components (as part of the intervention or for assessment), i.e., to what degree got the study team to know the participant. In online-only trials, clarify if participants were quasi-anonymous and whether having multiple identities was possible or whether technical or logistical measures (e.g., cookies, email confirmation, phone calls) were used to detect/prevent these.

	1	2	3	4	5	
subitem not at all important	○	○	○	○	◉	essential

Auswahl löschen

Does your paper address subitem 4a-ii? *

Copy and paste relevant sections from the manuscript (include quotes in quotation marks "like this" to indicate direct quotes from your manuscript), or elaborate on this item by providing additional information not in the ms, or briefly explain why the item is not applicable/relevant for your study

"All interactions and data collection occurred online via Prolific, an online recruitment platform. Participants initially underwent a screening process using an online questionnaire that collected information on demographics, lifestyle habits, mental health history, and access to technology. If they passed the screening, they were offered participation in the RCTs".
Researchers only had access to the Prolific ID and no other other personally identifiable information, which ensured participant privacy. The use of an established online recruitment platform like Prolific inherently reduces the risk of multiple identities due to their registration and verification processes

Ihre Antwort ist zu lang. Kürzen Sie einige Sätze in Ihrer Antwort.

4a-iii) Information giving during recruitment

Information given during recruitment. Specify how participants were briefed for recruitment and in the informed consent procedures (e.g., publish the informed consent documentation as appendix, see also item X26), as this information may have an effect on user self-selection, user expectation and may also bias results.

	1	2	3	4	5	
subitem not at all important	○	○	○	○	○	essential

Does your paper address subitem 4a-iii?

Copy and paste relevant sections from the manuscript (include quotes in quotation marks "like this" to indicate direct quotes from your manuscript), or elaborate on this item by providing additional information not in the ms, or briefly explain why the item is not applicable/relevant for your study

Meine Antwort

4b) Settings and locations where the data were collected

Does your paper address CONSORT subitem 4b? *

Copy and paste relevant sections from the manuscript (include quotes in quotation marks "like this" to indicate direct quotes from your manuscript), or elaborate on this item by providing additional information not in the ms, or briefly explain why the item is not applicable/relevant for your study

Data were collected online.

Ihre Antwort ist zu lang. Kürzen Sie einige Sätze in Ihrer Antwort.

4b-i) Report if outcomes were (self-)assessed through online questionnaires

Clearly report if outcomes were (self-)assessed through online questionnaires (as common in web-based trials) or otherwise.

	1	2	3	4	5	
subitem not at all important	○	○	○	○	◉	essential

Auswahl löschen

Ihre Antwort ist zu lang. Kürzen Sie einige Sätze in Ihrer Antwort.

Does your paper address subitem 4b-i? *

Copy and paste relevant sections from the manuscript (include quotes in quotation marks "like this" to indicate direct quotes from your manuscript), or elaborate on this item by providing additional information not in the ms, or briefly explain why the item is not applicable/relevant for your study

Outcomes were assessed through self-reported online questionnaires.

"Safety was monitored through items in the weekly surveys that asked participants to report any new serious adverse effects experienced in the past week. The Intervention group was asked “Have you experienced any negative effects from using the Alena app? This could be a physical or emotional effect that you believe you have experienced as a result of using the app and/or engaging in the app therapy.” Both groups were asked:
“Have you experienced any new, serious negative health effects in the past week? This includes having to see your GP for a new reason, going to hospital, or being otherwise very unwell in terms of your physical or mental health.” If participants responded positively to either question, they were prompted for additional details and to rate the severity of the event. Any reported events were reviewed by a clinician who determined whether the effect matched criteria for a “Serious Adverse Event”, as defined by the ISO 14155 (A:14).
Acceptability was assessed using custom-built questionnaires. Participants were asked how satisfied they were with the app overall (5-point Likert scale from very dissatisfied to very satisfied); how helpful they found the app (5-point Likert scale from very unhelpful to very helpful); how likely they would be to recommend the app (5-point Likert scale from very unlikely to very likely); how easy they found using the app (5-point Likert scale from very difficult to very easy); whether they got to the end of the weekly exercise (yes/no), and what got in the way of completing the exercises, with options provided. Furthermore, adherence to the therapy (monitored by in-app event markers) was monitored through participants' engagement with the app.
Symptoms were measured using the Social Phobia Inventory (SPIN [33]). Designed to evaluate the comprehensive range of symptoms associated with social anxiety – such as fear, avoidance, and physiological reactions – the SPIN includes 17 items, each scored from 0 to 4. This scoring system yields a total possible score ranging from 0 to 68. A score higher than 19 separates individuals with social anxiety from non-anxious controls [33,34]. A decrease of 10 points or more from the baseline SPIN score is considered a reliable indicator of significant improvement in social anxiety, according to the Reliable Change Index provided by The National Collaborating Centre for Mental Health (2018). A score ≤ 19 corresponds to subclinical levels of anxiety.
Daily functioning was assessed using the Work and Social Adjustment Scale (WSAS [35]). The WSAS evaluates how much a respondent's issue affects their ability to perform everyday tasks, including work, managing home responsibilities, and engaging in social and leisure activities. Each activity is rated on a scale ranging from 0 ("Not at all") to 8 ("Very severely"), with total scores ranging from 0 to 40."

Ihre Antwort ist zu lang. Kürzen Sie einige Sätze in Ihrer Antwort.

4b-ii) Report how institutional affiliations are displayed

Report how institutional affiliations are displayed to potential participants [on ehealth media], as affiliations with prestigious hospitals or universities may affect volunteer rates, use, and reactions with regards to an intervention.(Not a required item – describe only if this may bias results)

	1	2	3	4	5	
subitem not at all important	○	○	○	○	○	essential

Does your paper address subitem 4b-ii?

Copy and paste relevant sections from the manuscript (include quotes in quotation marks "like this" to indicate direct quotes from your manuscript), or elaborate on this item by providing additional information not in the ms, or briefly explain why the item is not applicable/relevant for your study

The study was conducted by Alena, a private company, which was clearly communicated to participants. This may have biased results.

5) The interventions for each group with sufficient details to allow replication, including how and when they were actually administered

5-i) Mention names, credential, affiliations of the developers, sponsors, and owners

Mention names, credential, affiliations of the developers, sponsors, and owners [6] (if authors/evaluators are owners or developer of the software, this needs to be declared in a “Conflict of interest” section or mentioned elsewhere in the manuscript).

	1	2	3	4	5	
subitem not at all important	○	○	○	○	○	essential

Ihre Antwort ist zu lang. Kürzen Sie einige Sätze in Ihrer Antwort.

Does your paper address subitem 5-i?

Copy and paste relevant sections from the manuscript (include quotes in quotation marks "like this" to indicate direct quotes from your manuscript), or elaborate on this item by providing additional information not in the ms, or briefly explain why the item is not applicable/relevant for your study

"Study authors MMG, JM, TM, SSM, SS, PS and AL were employed by Alena at the time of conducting the study and SL was paid on a consultancy basis. MMG, JM, SSM, SS, AL and MA own share options. QJMH is employed by University College London and acknowledges research grant funding from the Wellcome Trust, Carigest S.A. and Koa Health, and fees and share options for consultancies for Aya Technologies Ltd and Alto Neuroscience."

5-ii) Describe the history/development process

Describe the history/development process of the application and previous formative evaluations (e.g., focus groups, usability testing), as these will have an impact on adoption/use rates and help with interpreting results.

	1	2	3	4	5	
subitem not at all important	○	○	○	○	○	essential

Does your paper address subitem 5-ii?

Copy and paste relevant sections from the manuscript (include quotes in quotation marks "like this" to indicate direct quotes from your manuscript), or elaborate on this item by providing additional information not in the ms, or briefly explain why the item is not applicable/relevant for your study

"During the design of the app, detailed attention was paid to user design, including an appealing visual design, easy-to-use interface, bite-sized therapeutic content that was adapted to the participant population by providing relevant and normalizing examples."

Ihre Antwort ist zu lang. Kürzen Sie einige Sätze in Ihrer Antwort.

5-iii) Revisions and updating

Revisions and updating. Clearly mention the date and/or version number of the application/intervention (and comparator, if applicable) evaluated, or describe whether the intervention underwent major changes during the evaluation process, or whether the development and/or content was “frozen” during the trial. Describe dynamic components such as news feeds or changing content which may have an impact on the replicability of the intervention (for unexpected events see item 3b).

	1	2	3	4	5	
subitem not at all important	○	○	◉	○	○	essential

Auswahl löschen

Does your paper address subitem 5-iii?

Copy and paste relevant sections from the manuscript (include quotes in quotation marks "like this" to indicate direct quotes from your manuscript), or elaborate on this item by providing additional information not in the ms, or briefly explain why the item is not applicable/relevant for your study

The content was frozen during the trial.

5-iv) Quality assurance methods

Provide information on quality assurance methods to ensure accuracy and quality of information provided [1], if applicable.

	1	2	3	4	5	
subitem not at all important	○	○	◉	○	○	essential

Auswahl löschen

Ihre Antwort ist zu lang. Kürzen Sie einige Sätze in Ihrer Antwort.

Does your paper address subitem 5-iv?

Copy and paste relevant sections from the manuscript (include quotes in quotation marks "like this" to indicate direct quotes from your manuscript), or elaborate on this item by providing additional information not in the ms, or briefly explain why the item is not applicable/relevant for your study

Meine Antwort

5-v) Ensure replicability by publishing the source code, and/or providing screenshots/screen-capture video, and/or providing flowcharts of the algorithms used

Ensure replicability by publishing the source code, and/or providing screenshots/screen-capture video, and/or providing flowcharts of the algorithms used. Replicability (i.e., other researchers should in principle be able to replicate the study) is a hallmark of scientific reporting.

	1	2	3	4	5	
subitem not at all important	○	○	○	○	○	essential

Does your paper address subitem 5-v?

Copy and paste relevant sections from the manuscript (include quotes in quotation marks "like this" to indicate direct quotes from your manuscript), or elaborate on this item by providing additional information not in the ms, or briefly explain why the item is not applicable/relevant for your study

Meine Antwort

Ihre Antwort ist zu lang. Kürzen Sie einige Sätze in Ihrer Antwort.

5-vi) Digital preservation

Digital preservation: Provide the URL of the application, but as the intervention is likely to change or disappear over the course of the years; also make sure the intervention is archived (Internet Archive, webcitation.org, and/or publishing the source code or screenshots/videos alongside the article). As pages behind login screens cannot be archived, consider creating demo pages which are accessible without login.

	1	2	3	4	5	
subitem not at all important	○	○	○	○	○	essential

Does your paper address subitem 5-vi?

Copy and paste relevant sections from the manuscript (include quotes in quotation marks "like this" to indicate direct quotes from your manuscript), or elaborate on this item by providing additional information not in the ms, or briefly explain why the item is not applicable/relevant for your study

Meine Antwort

5-vii) Access

Access: Describe how participants accessed the application, in what setting/context, if they had to pay (or were paid) or not, whether they had to be a member of specific group. If known, describe how participants obtained "access to the platform and Internet" [1]. To ensure access for editors/reviewers/readers, consider to provide a "backdoor" login account or demo mode for reviewers/readers to explore the application (also important for archiving purposes, see vi).

	1	2	3	4	5	
subitem not at all important	○	○	○	○	○	essential

Ihre Antwort ist zu lang. Kürzen Sie einige Sätze in Ihrer Antwort.

Does your paper address subitem 5-vii? *

Copy and paste relevant sections from the manuscript (include quotes in quotation marks "like this" to indicate direct quotes from your manuscript), or elaborate on this item by providing additional information not in the ms, or briefly explain why the item is not applicable/relevant for your study

Participants accessed the application through a smartphone app. They received instructions on how to download and use the Alena app after being assigned to the intervention group following the screening process. The app was designed to be user-friendly, and participants were provided with guidance to ensure they could navigate the app effectively.
All interactions and data collection occurred online, with participants completing weekly assessments through the app in their own chosen environments. This was a fully web-based study, with no face-to-face components involved.

"The engagement with the app and the therapeutic content itself was not incentivised. However, participants received £1 for their involvement in the screening process. Furthermore, all participants in both RCTs were compensated £5 per survey independent of engagement with the app or therapy."

There was no requirement for participants to be members of a specific group. Eligibility was determined through the initial screening process conducted via Prolific.

Participants needed to have access to a smartphone with internet capability. Specifically:
For RCT #1: "smartphone with iOS and internet access"
For RCT #2: "smartphone with internet access and android or iOS operating system"

Ihre Antwort ist zu lang. Kürzen Sie einige Sätze in Ihrer Antwort.

5-viii) Mode of delivery, features/functionalities/components of the intervention and comparator, and the theoretical framework

Describe mode of delivery, features/functionalities/components of the intervention and comparator, and the theoretical framework [6] used to design them (instructional strategy [1], behaviour change techniques, persuasive features, etc., see e.g., [7, 8] for terminology). This includes an in-depth description of the content (including where it is coming from and who developed it) [1],” whether [and how] it is tailored to individual circumstances and allows users to track their progress and receive feedback” [6]. This also includes a description of communication delivery channels and – if computer-mediated communication is a component – whether communication was synchronous or asynchronous [6]. It also includes information on presentation strategies [1], including page design principles, average amount of text on pages, presence of hyperlinks to other resources, etc. [1].

	1	2	3	4	5	
subitem not at all important	○	○	◉	○	○	essential

Auswahl löschen

Ihre Antwort ist zu lang. Kürzen Sie einige Sätze in Ihrer Antwort.

Does your paper address subitem 5-viii? *

Copy and paste relevant sections from the manuscript (include quotes in quotation marks "like this" to indicate direct quotes from your manuscript), or elaborate on this item by providing additional information not in the ms, or briefly explain why the item is not applicable/relevant for your study

Ihre Antwort ist zu lang. Kürzen Sie einige Sätze in Ihrer Antwort.

Modules:

"Introduction: Serves as an introductory overview, setting the stage for the program and providing insight into the drivers of social anxiety symptoms."
"Beliefs: Focuses on conditional beliefs about oneself and others."
"Attention: Concentrates on self-awareness and self-focus during social interactions."
"Avoidance: Deals with safety behaviors and avoidance patterns."
"Rumination: Addresses the tendency to overthink or analyze social interactions after they occur" .

Recharge and Community Tabs (RCT #2):

"The Recharge section contained mindfulness-based exercises, guided meditations, journaling, and self-compassion exercises."
"The anonymous community provided an opportunity to connect with and get support from others by posting in an online chat forum" .

Game-like Assessments (RCT #2):

Introduced more engaging content, including "game-like assessments to engage participants further and assess their cognitive and behavioral patterns related to social anxiety. These assessments, lasting between 5 and 15 minutes, were positioned at the start of each module, and completion was required to unlock the rest of the exercises within that module."

The program was designed based on the "CBT competencies framework," integrating principles from Clark and Wells' therapy for social anxiety disorder (SAD). The framework targeted specific cognitive processes and mechanisms involved in SAD, such as negative beliefs, self-focused attention, rumination, and avoidance behaviors.

Instructional Strategy:

The app utilized "psychoeducational audio lessons and practical worksheets" to guide participants through the therapeutic content.

Techniques included cognitive restructuring (challenging negative beliefs), exposure exercises (gradual engagement with feared social situations), mindfulness practices, and self-compassion exercises to promote emotional regulation and reduce anxiety symptoms.

Persuasive Features:

The app featured "an appealing visual design, easy-to-use interface, and bite-sized therapeutic content" to enhance user engagement and adherence.

Content Origin and Development:

The content was developed by Alena, incorporating evidence-based CBT techniques adapted for digital delivery. The content aimed to address core components of social anxiety through [illegible]

Ihre Antwort ist zu lang. Kürzen Sie einige Sätze in Ihrer Antwort.

Tailoring:

The app allowed for some degree of personalization by enabling users to engage with modules at their own pace. In RCT #2, all modules were accessible from the start, allowing participants to select the most relevant modules based on their individual needs. Additionally, the Recharge and Community features provided supplementary support tailored to individual preferences .

Tracking Progress and Feedback:

Participants could track their progress through in-app event markers and completion of exercises. Weekly assessments using the Social Phobia Inventory (SPIN) and Work and Social Adjustment Scale (WSAS) provided regular feedback on symptom reduction and functional improvement.

Communication Delivery Channels:

The app primarily used asynchronous communication, with participants completing assessments and exercises at their convenience. The community forum allowed for asynchronous interactions among participants.

Presentation Strategies:

The app featured a clean and user-friendly interface with modules and exercises clearly categorized. Visual elements were designed to be engaging and easy to navigate .

Average Amount of Text and Hyperlinks:

The content was presented in bite-sized formats to prevent cognitive overload. Each module contained concise audio lessons and worksheets, with minimal text on each page. The presence of hyperlinks to additional resources was not explicitly mentioned, but the app design aimed to keep users focused on the primary therapeutic content .

5-ix) Describe use parameters

Describe use parameters (e.g., intended “doses” and optimal timing for use). Clarify what instructions or recommendations were given to the user, e.g., regarding timing, frequency, heaviness of use, if any, or was the intervention used ad libitum.

	1	2	3	4	5	
subitem not at all important	○	○	○	○	○	essential

Ihre Antwort ist zu lang. Kürzen Sie einige Sätze in Ihrer Antwort.

Does your paper address subitem 5-ix?

Copy and paste relevant sections from the manuscript (include quotes in quotation marks "like this" to indicate direct quotes from your manuscript), or elaborate on this item by providing additional information not in the ms, or briefly explain why the item is not applicable/relevant for your study

"The exercises, each taking between 1 to 8 minutes to complete, were designed to fit into the user's daily routine. The app encouraged participants to repeat exercises if needed and to extend their learning outside the app through real-life exposure experiments, supported by in-app exercises that assisted participants with planning and reflecting on these experiments.
To optimise the learning curve and ensure a structured progression through the program, the availability of modules was controlled. In RCT #1, modules were sequentially unlocked each week, while in RCT #2, all modules were accessible from the start, but participants were advised to complete one module every two weeks. To complete all recommended content in the app, participants would have needed to spend between 10 and 20 minutes on the app per week."

5-x) Clarify the level of human involvement

Clarify the level of human involvement (care providers or health professionals, also technical assistance) in the e-intervention or as co-intervention (detail number and expertise of professionals involved, if any, as well as "type of assistance offered, the timing and frequency of the support, how it is initiated, and the medium by which the assistance is delivered". It may be necessary to distinguish between the level of human involvement required for the trial, and the level of human involvement required for a routine application outside of a RCT setting (discuss under item 21 – generalizability).

	1	2	3	4	5	
subitem not at all important	○	○	○	○	○	essential

Ihre Antwort ist zu lang. Kürzen Sie einige Sätze in Ihrer Antwort.

Does your paper address subitem 5-x?

Copy and paste relevant sections from the manuscript (include quotes in quotation marks "like this" to indicate direct quotes from your manuscript), or elaborate on this item by providing additional information not in the ms, or briefly explain why the item is not applicable/relevant for your study

The intervention was designed to be entirely digital with no involvement of care providers or health professionals during the trial. The design of the study, with its lack of human involvement, reflects the intended real-world use of the app.

Technical assistance was provided when participants encountered technical problems. Participants could contact the technical support team if they faced any issues with accessing or using the app.

5-xi) Report any prompts/reminders used

Report any prompts/reminders used: Clarify if there were prompts (letters, emails, phone calls, SMS) to use the application, what triggered them, frequency etc. It may be necessary to distinguish between the level of prompts/reminders required for the trial, and the level of prompts/reminders for a routine application outside of a RCT setting (discuss under item 21 – generalizability).

	1	2	3	4	5	
subitem not at all important	○	○	○	○	○	essential

Does your paper address subitem 5-xi? *

Copy and paste relevant sections from the manuscript (include quotes in quotation marks "like this" to indicate direct quotes from your manuscript), or elaborate on this item by providing additional information not in the ms, or briefly explain why the item is not applicable/relevant for your study

Participants were reminded weekly via Prolific message to fill in the questionnaire.

Ihre Antwort ist zu lang. Kürzen Sie einige Sätze in Ihrer Antwort.

5-xii) Describe any co-interventions (incl. training/support)

Describe any co-interventions (incl. training/support): Clearly state any interventions that are provided in addition to the targeted eHealth intervention, as ehealth intervention may not be designed as stand-alone intervention. This includes training sessions and support [1]. It may be necessary to distinguish between the level of training required for the trial, and the level of training for a routine application outside of a RCT setting (discuss under item 21 – generalizability.

	1	2	3	4	5	
subitem not at all important	○	○	○	○	○	essential

Does your paper address subitem 5-xii? *

Copy and paste relevant sections from the manuscript (include quotes in quotation marks "like this" to indicate direct quotes from your manuscript), or elaborate on this item by providing additional information not in the ms, or briefly explain why the item is not applicable/relevant for your study

"Participants in RCT #2 additionally had access to a Recharge and a Community section in the app (Figure 2B). The Recharge section contained mindfulness-based exercises, guided meditations, journaling and self-compassion exercises designed to help participants overcome negative thoughts and feelings related to social situations. For example, participants learned how to observe thoughts without automatically identifying with them, wrote a compassionate letter to the self and were encouraged to celebrate a small win. The anonymous community provided an opportunity to connect with and get support from others by posting in an online chat forum. Participants were suggested to try one Recharge exercise per week and engage with a Community post in some way each week (by liking and/or commenting, and considering posting if they feel comfortable)."

6a) Completely defined pre-specified primary and secondary outcome measures, including how and when they were assessed

Ihre Antwort ist zu lang. Kürzen Sie einige Sätze in Ihrer Antwort.

Does your paper address CONSORT subitem 6a? *

Copy and paste relevant sections from the manuscript (include quotes in quotation marks "like this" to indicate direct quotes from your manuscript), or elaborate on this item by providing additional information not in the ms, or briefly explain why the item is not applicable/relevant for your study

"RCT #1: Primary outcome measures were safety and acceptability. Secondary outcome measures were symptoms and functioning at week 4 and two weeks post-intervention at week 6.
RCT #2: Primary outcome measures were change in symptoms and daily functioning from baseline to week 8. Secondary outcome measures were safety, efficacy and daily functioning four weeks post-intervention at week 12.

"Safety was monitored through items in the weekly surveys that asked participants to report any new serious adverse effects experienced in the past week."
"Acceptability was assessed using custom-built questionnaires."
"Symptoms were measured using the Social Phobia Inventory (SPIN [33])."
"Daily functioning was assessed using the Work and Social Adjustment Scale (WSAS [35])."

See reply to 4b-i for detailed information about the questionnaires.

6a-i) Online questionnaires: describe if they were validated for online use and apply CHERRIES items to describe how the questionnaires were designed/deployed

If outcomes were obtained through online questionnaires, describe if they were validated for online use and apply CHERRIES items to describe how the questionnaires were designed/deployed [9].

	1	2	3	4	5	
subitem not at all important	○	○	○	○	○	essential

Does your paper address subitem 6a-i?

Copy and paste relevant sections from manuscript text

The Social Phobia Inventory (SPIN) and the Work and Social Adjustment Scale (WSAS) were used for outcome assessments. Both scales are widely recognized and have been validated for online use.

Ihre Antwort ist zu lang. Kürzen Sie einige Sätze in Ihrer Antwort.

6a-ii) Describe whether and how “use” (including intensity of use/dosage) was defined/measured/monitored

Describe whether and how “use” (including intensity of use/dosage) was defined/measured/monitored (logins, logfile analysis, etc.). Use/adoption metrics are important process outcomes that should be reported in any ehealth trial.

	1	2	3	4	5	
subitem not at all important	○	○	○	○	○	essential

Does your paper address subitem 6a-ii?

Copy and paste relevant sections from manuscript text

Therapy adherence was monitored through in-app event markers, which tracked the number of audio lessons listened to and interactive worksheets finished by each participant.

"Furthermore, adherence to the therapy (monitored by in-app event markers) was monitored through participants' engagement with the app."

For RCT #1, the median completion rate was 90.91% (mean = 76.92%, SD = 29.17%).
For RCT #2, the median completion rate was 84.85% (mean = 73.83%, SD = 27.89%) ."

6a-iii) Describe whether, how, and when qualitative feedback from participants was obtained

Describe whether, how, and when qualitative feedback from participants was obtained (e.g., through emails, feedback forms, interviews, focus groups).

	1	2	3	4	5	
subitem not at all important	○	○	○	○	○	essential

Ihre Antwort ist zu lang. Kürzen Sie einige Sätze in Ihrer Antwort.

Does your paper address subitem 6a-iii?

Copy and paste relevant sections from manuscript text

Qualitative feedback from users was obtained during the development of the app (focus groups, qualitative questionnaires).

During the RCTs, qualitative feedback from participants was obtained through custom-built questionnaires. These questionnaires included items specifically designed to gather subjective feedback on various aspects of the participants' experiences with the Alena app.

"Participants were asked how satisfied they were with the app overall [...]; how helpful they found the app [...]; how likely they would be to recommend the app [...]; how easy they found using the app [...]; whether they got to the end of the weekly exercise (yes/no), and what got in the way of completing the exercises, with options provided." (See reply to 4b-i for details).

Additionally, participants were asked about their engagement with the weekly exercises, including whether they completed the exercises and what obstacles they faced if they did not complete them .

6b) Any changes to trial outcomes after the trial commenced, with reasons

Does your paper address CONSORT subitem 6b? *

Copy and paste relevant sections from the manuscript (include quotes in quotation marks "like this" to indicate direct quotes from your manuscript), or elaborate on this item by providing additional information not in the ms, or briefly explain why the item is not applicable/relevant for your study

No changes to trial outcomes after the trial commenced.

7a) How sample size was determined

NPT: When applicable, details of whether and how the clustering by care provides or centers was addressed

Ihre Antwort ist zu lang. Kürzen Sie einige Sätze in Ihrer Antwort.

7a-i) Describe whether and how expected attrition was taken into account when calculating the sample size

Describe whether and how expected attrition was taken into account when calculating the sample size.

	1	2	3	4	5	
subitem not at all important	○	○	○	○	○	essential

Does your paper address subitem 7a-i?

Copy and paste relevant sections from manuscript title (include quotes in quotation marks "like this" to indicate direct quotes from your manuscript), or elaborate on this item by providing additional information not in the ms, or briefly explain why the item is not applicable/relevant for your study

"Based on a G*Power analysis for the difference between the groups (two-sided t-test), with an estimated medium effect size (d) of 0.6, an alpha level of 0.05, and 80% power, the required sample size is 45 participants per group. Considering a 10% likelihood of participant dropout, we increased our target sample size to 50 participants per group. Sample sizes for RCT#2 were based on effect size estimates from RCT #1 (Cohen's d of 0.47 after 4 weeks). To detect effect sizes of 0.47 with an alpha level of 0.05, and a power of 95%, a sample size of 119 participants per group is required. Considering the likelihood of participant dropout, we increased our target sample size to 125 participants per group."

7b) When applicable, explanation of any interim analyses and stopping guidelines

Does your paper address CONSORT subitem 7b? *

Copy and paste relevant sections from the manuscript (include quotes in quotation marks "like this" to indicate direct quotes from your manuscript), or elaborate on this item by providing additional information not in the ms, or briefly explain why the item is not applicable/relevant for your study

No interim analyses / stopping guidelines.

Ihre Antwort ist zu lang. Kürzen Sie einige Sätze in Ihrer Antwort.

Does your paper address CONSORT subitem 8a? *

Copy and paste relevant sections from the manuscript (include quotes in quotation marks "like this" to indicate direct quotes from your manuscript), or elaborate on this item by providing additional information not in the ms, or briefly explain why the item is not applicable/relevant for your study

No care providers were allocated.

8b) Type of randomisation; details of any restriction (such as blocking and block size)

Does your paper address CONSORT subitem 8b? *

Copy and paste relevant sections from the manuscript (include quotes in quotation marks "like this" to indicate direct quotes from your manuscript), or elaborate on this item by providing additional information not in the ms, or briefly explain why the item is not applicable/relevant for your study

"In both trials, participants were randomized 1:1 to the active arm with access to the smartphone app, or the control arm without access to the smartphone app."

9) Mechanism used to implement the random allocation sequence (such as sequentially numbered containers), describing any steps taken to conceal the sequence until interventions were assigned

Does your paper address CONSORT subitem 9? *

Copy and paste relevant sections from the manuscript (include quotes in quotation marks "like this" to indicate direct quotes from your manuscript), or elaborate on this item by providing additional information not in the ms, or briefly explain why the item is not applicable/relevant for your study

We set up a custom-built randomisation code.

Ihre Antwort ist zu lang. Kürzen Sie einige Sätze in Ihrer Antwort.

10) Who generated the random allocation sequence, who enrolled participants, and who assigned participants to interventions

Does your paper address CONSORT subitem 10? *

Copy and paste relevant sections from the manuscript (include quotes in quotation marks "like this" to indicate direct quotes from your manuscript), or elaborate on this item by providing additional information not in the ms, or briefly explain why the item is not applicable/relevant for your study

The random allocation code was built by an engineer on the team. Participants were assigned randomly to the intervention.
"Participants initially underwent a screening process using an online questionnaire that collected information on demographics, lifestyle habits, mental health history, and access to technology. If they passed the screening, they were offered participation in the RCTs."

11a) If done, who was blinded after assignment to interventions (for example, participants, care providers, those assessing outcomes) and how

NPT: Whether or not administering co-interventions were blinded to group assignment

11a-i) Specify who was blinded, and who wasn't

Specify who was blinded, and who wasn't. Usually, in web-based trials it is not possible to blind the participants [1, 3] (this should be clearly acknowledged), but it may be possible to blind outcome assessors, those doing data analysis or those administering co-interventions (if any).

	1	2	3	4	5	
subitem not at all important	○	○	○	○	○	essential

Ihre Antwort ist zu lang. Kürzen Sie einige Sätze in Ihrer Antwort.

Does your paper address subitem 11a-i? *

Copy and paste relevant sections from the manuscript (include quotes in quotation marks "like this" to indicate direct quotes from your manuscript), or elaborate on this item by providing additional information not in the ms, or briefly explain why the item is not applicable/relevant for your study

Neither participants nor researchers were blinded.
"We conducted two web-based, unblinded, randomized controlled trials (RCTs)" .

11a-ii) Discuss e.g., whether participants knew which intervention was the "intervention of interest" and which one was the "comparator"

Informed consent procedures (4a-ii) can create biases and certain expectations - discuss e.g., whether participants knew which intervention was the "intervention of interest" and which one was the "comparator".

	1	2	3	4	5	
subitem not at all important	○	○	○	○	○	essential

Does your paper address subitem 11a-ii?

Copy and paste relevant sections from the manuscript (include quotes in quotation marks "like this" to indicate direct quotes from your manuscript), or elaborate on this item by providing additional information not in the ms, or briefly explain why the item is not applicable/relevant for your study

Participants were aware of whether they had access to the treatment or not.

11b) If relevant, description of the similarity of interventions

(this item is usually not relevant for ehealth trials as it refers to similarity of a placebo or sham intervention to a active medication/intervention)

Ihre Antwort ist zu lang. Kürzen Sie einige Sätze in Ihrer Antwort.

Does your paper address CONSORT subitem 11b? *

Copy and paste relevant sections from the manuscript (include quotes in quotation marks "like this" to indicate direct quotes from your manuscript), or elaborate on this item by providing additional information not in the ms, or briefly explain why the item is not applicable/relevant for your study

This item is not relevant.

12a) Statistical methods used to compare groups for primary and secondary outcomes

NPT: When applicable, details of whether and how the clustering by care providers or centers was addressed

Does your paper address CONSORT subitem 12a? *

Copy and paste relevant sections from the manuscript (include quotes in quotation marks "like this" to indicate direct quotes from your manuscript), or elaborate on this item by providing additional information not in the ms, or briefly explain why the item is not applicable/relevant for your study

"Safety: We compared the number of adverse events between groups using a χ^2-test.
Acceptability: We characterized acceptability descriptively. Dropout rates were also compared using a χ^2-test.
Efficacy and daily functioning: Planned intention-to-treat analyses were performed to compare SPIN (efficacy) and WSAS (daily functioning) scores across groups. Specifically, we used independent t-tests to compare mean changes in scores from baseline to the end of the intervention period, and from the end of the intervention period to the follow-up, between the intervention and waitlist control groups. We used the Benjamini-Hochberg method of False Discovery Rate (FDR) correction to correct for multiple comparisons. Chi-squared tests were used to analyze categorical outcomes across groups, such as the proportion of participants who achieved a clinically significant improvement in social anxiety symptoms (defined as a reduction of ≥10 points in SPIN scores) and those who reached subclinical levels of symptoms (SPIN ≤ 19)."

Ihre Antwort ist zu lang. Kürzen Sie einige Sätze in Ihrer Antwort.

12a-i) Imputation techniques to deal with attrition / missing values

Imputation techniques to deal with attrition / missing values: Not all participants will use the intervention/comparator as intended and attrition is typically high in ehealth trials. Specify how participants who did not use the application or dropped out from the trial were treated in the statistical analysis (a complete case analysis is strongly discouraged, and simple imputation techniques such as LOCF may also be problematic [4]).

	1	2	3	4	5	
subitem not at all important	○	○	○	○	○	essential

Does your paper address subitem 12a-i? *

Copy and paste relevant sections from the manuscript (include quotes in quotation marks "like this" to indicate direct quotes from your manuscript), or elaborate on this item by providing additional information not in the ms, or briefly explain why the item is not applicable/relevant for your study

"Planned intention-to-treat analyses were performed to compare SPIN (efficacy) and WSAS (daily functioning) scores across groups".
This approach ensures that all participants originally allocated to the intervention and control groups are included in the analysis, regardless of whether they completed the intervention or dropped out.

12b) Methods for additional analyses, such as subgroup analyses and adjusted analyses

Ihre Antwort ist zu lang. Kürzen Sie einige Sätze in Ihrer Antwort.

Does your paper address CONSORT subitem 12b? *

Copy and paste relevant sections from the manuscript (include quotes in quotation marks "like this" to indicate direct quotes from your manuscript), or elaborate on this item by providing additional information not in the ms, or briefly explain why the item is not applicable/relevant for your study

"In an exploratory analysis, linear mixed-effects regression modeling was implemented to evaluate the change in SPIN and WSAS scores over time. The models included fixed effects for age, group, time (week), a quadratic time effect (week2) accounting for non-linear change in scores over time such as plateau effects and a group × time interaction to assess differential changes in scores between the intervention and control groups across the study period. In RCT #2, additional fixed effects included sex, a group x sex, and a group x week x sex interaction to examine differential changes in scores by sex. Furthermore, the model included a random intercept for each participant, accounting for the baseline variability in scores among individuals."

X26) REB/IRB Approval and Ethical Considerations [recommended as subheading under "Methods"] (not a CONSORT item)

X26-i) Comment on ethics committee approval

	1	2	3	4	5	
subitem not at all important	○	○	○	○	○	essential

Does your paper address subitem X26-i?

Copy and paste relevant sections from the manuscript (include quotes in quotation marks "like this" to indicate direct quotes from your manuscript), or elaborate on this item by providing additional information not in the ms, or briefly explain why the item is not applicable/relevant for your study

"The studies received approval from the Reading Independent Ethics Committee (study reference: AYSATOL)."

Ihre Antwort ist zu lang. Kürzen Sie einige Sätze in Ihrer Antwort.

x26-ii) Outline informed consent procedures

Outline informed consent procedures e.g., if consent was obtained offline or online (how? Checkbox, etc.?), and what information was provided (see 4a-ii). See [6] for some items to be included in informed consent documents.

	1	2	3	4	5	
subitem not at all important	○	○	○	○	○	essential

Does your paper address subitem X26-ii?

Copy and paste relevant sections from the manuscript (include quotes in quotation marks "like this" to indicate direct quotes from your manuscript), or elaborate on this item by providing additional information not in the ms, or briefly explain why the item is not applicable/relevant for your study

"Participants provided informed consent digitally before engaging in any part of the study."

X26-iii) Safety and security procedures

Safety and security procedures, incl. privacy considerations, and any steps taken to reduce the likelihood or detection of harm (e.g., education and training, availability of a hotline)

	1	2	3	4	5	
subitem not at all important	○	○	○	○	○	essential

Does your paper address subitem X26-iii?

Copy and paste relevant sections from the manuscript (include quotes in quotation marks "like this" to indicate direct quotes from your manuscript), or elaborate on this item by providing additional information not in the ms, or briefly explain why the item is not applicable/relevant for your study

Meine Antwort

Ihre Antwort ist zu lang. Kürzen Sie einige Sätze in Ihrer Antwort.

13a) For each group, the numbers of participants who were randomly assigned, received intended treatment, and were analysed for the primary outcome
NPT: The number of care providers or centers performing the intervention in each group and the number of patients treated by each care provider in each center

Does your paper address CONSORT subitem 13a? *

Copy and paste relevant sections from the manuscript (include quotes in quotation marks "like this" to indicate direct quotes from your manuscript), or elaborate on this item by providing additional information not in the ms, or briefly explain why the item is not applicable/relevant for your study

"Eligible participants from the screening studies (45.14% in RCT #1 and 27.22% in RCT #2, Figure 2) were invited to participate in the main study. RCT #1 included a total of 102 participants (all female, aged between 18 and 35, 50 in intervention and 52 in control group). RCT #2 included 249 participants in total (64% females, 36% males, aged between 18 and 75). One participant in the intervention group of RCT #2 was excluded from the study due to reporting they no longer had access to a smartphone with Internet access in the baseline assessment, yielding N = 124 in the intervention group and N = 124 in the control group."

13b) For each group, losses and exclusions after randomisation, together with reasons

Does your paper address CONSORT subitem 13b? (NOTE: Preferably, this is shown in a CONSORT flow diagram) *

Copy and paste relevant sections from the manuscript (include quotes in quotation marks "like this" to indicate direct quotes from your manuscript), or elaborate on this item by providing additional information not in the ms, or briefly explain why the item is not applicable/relevant for your study

"One participant in the intervention group of RCT #2 was excluded from the study due to reporting they no longer had access to a smartphone with Internet access in the baseline assessment,"

Ihre Antwort ist zu lang. Kürzen Sie einige Sätze in Ihrer Antwort.

13b-i) Attrition diagram

Strongly recommended: An attrition diagram (e.g., proportion of participants still logging in or using the intervention/comparator in each group plotted over time, similar to a survival curve) or other figures or tables demonstrating usage/dose/engagement.

	1	2	3	4	5	
subitem not at all important	○	○	○	○	○	essential

Does your paper address subitem 13b-i?

Copy and paste relevant sections from the manuscript or cite the figure number if applicable (include quotes in quotation marks "like this" to indicate direct quotes from your manuscript), or elaborate on this item by providing additional information not in the ms, or briefly explain why the item is not applicable/relevant for your study

"Figure 2. Consort diagram. Flow of participants through the trial for RCT #1 (A) and RCT #2 (B)."
"Figure 5. Therapy completion rates."

14a) Dates defining the periods of recruitment and follow-up

Does your paper address CONSORT subitem 14a? *

Copy and paste relevant sections from the manuscript (include quotes in quotation marks "like this" to indicate direct quotes from your manuscript), or elaborate on this item by providing additional information not in the ms, or briefly explain why the item is not applicable/relevant for your study

"RCT #1 was a six-week parallel-group randomized controlled trial with a four-week intervention and a two-week follow-up. RCT #2 was an eight-week trial with a four-week follow-up."

Ihre Antwort ist zu lang. Kürzen Sie einige Sätze in Ihrer Antwort.

14a-i) Indicate if critical “secular events” fell into the study period

Indicate if critical “secular events” fell into the study period, e.g., significant changes in Internet resources available or “changes in computer hardware or Internet delivery resources”

	1	2	3	4	5	
subitem not at all important	○	○	○	○	○	essential

Does your paper address subitem 14a-i?

Copy and paste relevant sections from the manuscript (include quotes in quotation marks "like this" to indicate direct quotes from your manuscript), or elaborate on this item by providing additional information not in the ms, or briefly explain why the item is not applicable/relevant for your study

Meine Antwort

14b) Why the trial ended or was stopped (early)

Does your paper address CONSORT subitem 14b? *

Copy and paste relevant sections from the manuscript (include quotes in quotation marks "like this" to indicate direct quotes from your manuscript), or elaborate on this item by providing additional information not in the ms, or briefly explain why the item is not applicable/relevant for your study

The trial ended as planned and was not stopped early.

15) A table showing baseline demographic and clinical characteristics for each group

NPT: When applicable, a description of care providers (case volume, qualification, expertise, etc.) and centers (volume) in each group

Ihre Antwort ist zu lang. Kürzen Sie einige Sätze in Ihrer Antwort.

Does your paper address CONSORT subitem 15? *

Copy and paste relevant sections from the manuscript (include quotes in quotation marks "like this" to indicate direct quotes from your manuscript), or elaborate on this item by providing additional information not in the ms, or briefly explain why the item is not applicable/relevant for your study

"Table 1. Baseline characteristics of study participants."

15-i) Report demographics associated with digital divide issues

In ehealth trials it is particularly important to report demographics associated with digital divide issues, such as age, education, gender, social-economic status, computer/Internet/ehealth literacy of the participants, if known.

	1	2	3	4	5	
subitem not at all important	○	○	○	○	○	essential

Does your paper address subitem 15-i? *

Copy and paste relevant sections from the manuscript (include quotes in quotation marks "like this" to indicate direct quotes from your manuscript), or elaborate on this item by providing additional information not in the ms, or briefly explain why the item is not applicable/relevant for your study

"Participants initially underwent a screening process using an online questionnaire that collected information on demographics, lifestyle habits, mental health history, and access to technology." This included age, education, sex and experience with apps for mental health.

16) For each group, number of participants (denominator) included in each analysis and whether the analysis was by original assigned groups

Ihre Antwort ist zu lang. Kürzen Sie einige Sätze in Ihrer Antwort.

16-i) Report multiple “denominators” and provide definitions

Report multiple “denominators” and provide definitions: Report N’s (and effect sizes) “across a range of study participation [and use] thresholds” [1], e.g., N exposed, N consented, N used more than x times, N used more than y weeks, N participants “used” the intervention/comparator at specific pre-defined time points of interest (in absolute and relative numbers per group). Always clearly define “use” of the intervention.

	1	2	3	4	5	
subitem not at all important	○	○	○	○	○	essential

Does your paper address subitem 16-i? *

Copy and paste relevant sections from the manuscript (include quotes in quotation marks "like this" to indicate direct quotes from your manuscript), or elaborate on this item by providing additional information not in the ms, or briefly explain why the item is not applicable/relevant for your study

Number of participants are reported in each analysis.

16-ii) Primary analysis should be intent-to-treat

Primary analysis should be intent-to-treat, secondary analyses could include comparing only “users”, with the appropriate caveats that this is no longer a randomized sample (see 18-i).

	1	2	3	4	5	
subitem not at all important	○	○	○	○	○	essential

Does your paper address subitem 16-ii?

Copy and paste relevant sections from the manuscript (include quotes in quotation marks "like this" to indicate direct quotes from your manuscript), or elaborate on this item by providing additional information not in the ms, or briefly explain why the item is not applicable/relevant for your study

Primary analysis is intent-to-treat

Ihre Antwort ist zu lang. Kürzen Sie einige Sätze in Ihrer Antwort.

17a) For each primary and secondary outcome, results for each group, and the estimated effect size and its precision (such as 95% confidence interval)

Does your paper address CONSORT subitem 17a? *

Copy and paste relevant sections from the manuscript (include quotes in quotation marks "like this" to indicate direct quotes from your manuscript), or elaborate on this item by providing additional information not in the ms, or briefly explain why the item is not applicable/relevant for your study

Results are reported for each group, including mean ± standard deviation and effect size.
For example:
"By the end of the 4-week intervention period, participants in RCT #1 with access to the Alena app saw a significantly greater reduction in SPIN (-9.83 ± 12.80) compared to the waitlist control group (-4.13 ± 11.59, t90 = -2.23, pFDR = .037, Cohen's d = 0.47, Figure 5A, B)."

17a-i) Presentation of process outcomes such as metrics of use and intensity of use

In addition to primary/secondary (clinical) outcomes, the presentation of process outcomes such as metrics of use and intensity of use (dose, exposure) and their operational definitions is critical. This does not only refer to metrics of attrition (13-b) (often a binary variable), but also to more continuous exposure metrics such as “average session length”. These must be accompanied by a technical description how a metric like a “session” is defined (e.g., timeout after idle time) [1] (report under item 6a).

	1	2	3	4	5	
subitem not at all important	○	○	○	○	○	essential

Does your paper address subitem 17a-i?

Copy and paste relevant sections from the manuscript (include quotes in quotation marks "like this" to indicate direct quotes from your manuscript), or elaborate on this item by providing additional information not in the ms, or briefly explain why the item is not applicable/relevant for your study

Meine Antwort

Ihre Antwort ist zu lang. Kürzen Sie einige Sätze in Ihrer Antwort.

17b) For binary outcomes, presentation of both absolute and relative effect sizes is recommended

Does your paper address CONSORT subitem 17b? *

Copy and paste relevant sections from the manuscript (include quotes in quotation marks "like this" to indicate direct quotes from your manuscript), or elaborate on this item by providing additional information not in the ms, or briefly explain why the item is not applicable/relevant for your study

Both absolute and relative effect sizes are reported for binary outcomes, for example "Three participants allocated to the intervention group (6.0%) and eight participants allocated to the waitlist control group (16%) reported adverse effects at some point during the study ($\chi 2 = 1.81$; $p = .178$, Figure 3)"

18) Results of any other analyses performed, including subgroup analyses and adjusted analyses, distinguishing pre-specified from exploratory

Does your paper address CONSORT subitem 18? *

Copy and paste relevant sections from the manuscript (include quotes in quotation marks "like this" to indicate direct quotes from your manuscript), or elaborate on this item by providing additional information not in the ms, or briefly explain why the item is not applicable/relevant for your study

"In an exploratory analysis, linear mixed-effects regression modeling was implemented to evaluate the change in SPIN and WSAS scores over time."

18-i) Subgroup analysis of comparing only users

A subgroup analysis of comparing only users is not uncommon in ehealth trials, but if done, it must be stressed that this is a self-selected sample and no longer an unbiased sample from a randomized trial (see 16-iii).

	1	2	3	4	5	
subitem not at all important	○	○	○	○	○	essential

Ihre Antwort ist zu lang. Kürzen Sie einige Sätze in Ihrer Antwort.

Does your paper address subitem 18-i?

Copy and paste relevant sections from the manuscript (include quotes in quotation marks "like this" to indicate direct quotes from your manuscript), or elaborate on this item by providing additional information not in the ms, or briefly explain why the item is not applicable/relevant for your study

Meine Antwort

19) All important harms or unintended effects in each group
(for specific guidance see CONSORT for harms)

Does your paper address CONSORT subitem 19? *

Copy and paste relevant sections from the manuscript (include quotes in quotation marks "like this" to indicate direct quotes from your manuscript), or elaborate on this item by providing additional information not in the ms, or briefly explain why the item is not applicable/relevant for your study

"Adverse health events reported by both groups" are included in the Supplementary Material.

19-i) Include privacy breaches, technical problems

Include privacy breaches, technical problems. This does not only include physical "harm" to participants, but also incidents such as perceived or real privacy breaches [1], technical problems, and other unexpected/unintended incidents. "Unintended effects" also includes unintended positive effects [2].

	1	2	3	4	5	
subitem not at all important	○	○	○	○	○	essential

Ihre Antwort ist zu lang. Kürzen Sie einige Sätze in Ihrer Antwort.

Does your paper address subitem 19-i?

Copy and paste relevant sections from the manuscript (include quotes in quotation marks "like this" to indicate direct quotes from your manuscript), or elaborate on this item by providing additional information not in the ms, or briefly explain why the item is not applicable/relevant for your study

Meine Antwort

19-ii) Include qualitative feedback from participants or observations from staff/researchers

Include qualitative feedback from participants or observations from staff/researchers, if available, on strengths and shortcomings of the application, especially if they point to unintended/unexpected effects or uses. This includes (if available) reasons for why people did or did not use the application as intended by the developers.

	1	2	3	4	5	
subitem not at all important	○	○	○	○	○	essential

Does your paper address subitem 19-ii?

Copy and paste relevant sections from the manuscript (include quotes in quotation marks "like this" to indicate direct quotes from your manuscript), or elaborate on this item by providing additional information not in the ms, or briefly explain why the item is not applicable/relevant for your study

Meine Antwort

DISCUSSION

22) Interpretation consistent with results, balancing benefits and harms, and considering other relevant evidence

NPT: In addition, take into account the choice of the comparator, lack of or partial blinding, and unequal expertise of care providers or centers in each group

Ihre Antwort ist zu lang. Kürzen Sie einige Sätze in Ihrer Antwort.

22-i) Restate study questions and summarize the answers suggested by the data, starting with primary outcomes and process outcomes (use)

Restate study questions and summarize the answers suggested by the data, starting with primary outcomes and process outcomes (use).

	1	2	3	4	5	
subitem not at all important	○	○	○	○	○	essential

Does your paper address subitem 22-i? *

Copy and paste relevant sections from the manuscript (include quotes in quotation marks "like this" to indicate direct quotes from your manuscript), or elaborate on this item by providing additional information not in the ms, or briefly explain why the item is not applicable/relevant for your study

"Principal results
The findings from our two randomized controlled trials provide evidence for the efficacy, safety, and acceptability of an online-only, modularized iCBT program for SAD in the form of a smartphone app (alena.com).
Safety and acceptability
There were no indications that the online delivery of the interventions were unsafe. There was no increase in the severity or frequency of adverse events in the intervention group compared to the waitlist control group. The treatment included steps such as exposure which is crucial for therapeutic benefit but necessarily induces discomfort and could in principle be unsafe. The interventions made this very explicit, and provided instructions on how to engage in exposure safely. Hence, exposure can be delivered safely online without therapist involvement. This finding is also consistent with a recent meta-analysis that found that internet-based cognitive behavioral therapy (iCBT) is generally safer than control conditions [38].
Acceptability
During the design of the app, detailed attention was paid to user design, including an appealing visual design, easy-to-use interface, bite-sized therapeutic content that was adapted to the participant population by providing relevant and normalizing examples. Overall, this led to high acceptability scores and positive user feedback. The high completion rate of the program (users completed 84-91% of material in the app) suggests that these features helped effectively engage users, encouraging consistent participation and adherence to the treatment protocol. As such, the results speak to the importance of careful user interface design for online interventions.
Efficacy
The app demonstrated robust efficacy across both an unselected adult online sample, and in a sample of women aged 18-35. The latter group is particularly important given the high prevalence of social anxiety in younger women. In both groups, the positive treatment [illegible]

Ihre Antwort ist zu lang. Kürzen Sie einige Sätze in Ihrer Antwort.

22-ii) Highlight unanswered new questions, suggest future research

Highlight unanswered new questions, suggest future research.

	1	2	3	4	5	
subitem not at all important	○	○	○	○	○	essential

Does your paper address subitem 22-ii?

Copy and paste relevant sections from the manuscript (include quotes in quotation marks "like this" to indicate direct quotes from your manuscript), or elaborate on this item by providing additional information not in the ms, or briefly explain why the item is not applicable/relevant for your study

"Here, iCBT retained efficacy despite the modularized format and the absence of a therapist intervention. This opens new doors for treating SAD by encouraging users to focus on specific cognitive and behavioral processes most relevant to their individual needs. The ability to tailor treatment plans based on individual profiles may ultimately help address the heterogeneity in symptom presentation and treatment response among individuals with SAD [29]."

20) Trial limitations, addressing sources of potential bias, imprecision, and, if relevant, multiplicity of analyses

20-i) Typical limitations in ehealth trials

Typical limitations in ehealth trials: Participants in ehealth trials are rarely blinded. Ehealth trials often look at a multiplicity of outcomes, increasing risk for a Type I error. Discuss biases due to non-use of the intervention/usability issues, biases through informed consent procedures, unexpected events.

	1	2	3	4	5	
subitem not at all important	○	○	○	○	○	essential

Ihre Antwort ist zu lang. Kürzen Sie einige Sätze in Ihrer Antwort.

Does your paper address subitem 20-i? *

Copy and paste relevant sections from the manuscript (include quotes in quotation marks "like this" to indicate direct quotes from your manuscript), or elaborate on this item by providing additional information not in the ms, or briefly explain why the item is not applicable/relevant for your study

"There are several important limitations to consider. The first is the short follow-up time. [...]
The second limitation concerns the sample, which was limited to active user of the online recruitment website prolific.com. [...]
Third, we used a waitlist control as opposed to an active control condition as the comparison group in our study. [...]
Fourth, although the therapy content is modularized, the current studies the delivered interventions in a standardized fashion, with modules released in a fixed order.
Lastly, we cannot rule out that the efficacy in RCT #2 is due to engagement with the Recharge and Community content that participants in the intervention group had access to throughout the duration of the trial. [...]

21) Generalisability (external validity, applicability) of the trial findings

NPT: External validity of the trial findings according to the intervention, comparators, patients, and care providers or centers involved in the trial

21-i) Generalizability to other populations

Generalizability to other populations: In particular, discuss generalizability to a general Internet population, outside of a RCT setting, and general patient population, including applicability of the study results for other organizations

	1	2	3	4	5	
subitem not at all important	○	○	○	○	○	essential

Ihre Antwort ist zu lang. Kürzen Sie einige Sätze in Ihrer Antwort.

Does your paper address subitem 21-i?

Copy and paste relevant sections from the manuscript (include quotes in quotation marks "like this" to indicate direct quotes from your manuscript), or elaborate on this item by providing additional information not in the ms, or briefly explain why the item is not applicable/relevant for your study

"It is in particular not possible to generalize findings from a self-selected online panel of volunteers to a clinical sample. As such, the applicability of our results to real-world clinical populations will need to be assessed"

21-ii) Discuss if there were elements in the RCT that would be different in a routine application setting

Discuss if there were elements in the RCT that would be different in a routine application setting (e.g., prompts/reminders, more human involvement, training sessions or other co-interventions) and what impact the omission of these elements could have on use, adoption, or outcomes if the intervention is applied outside of a RCT setting.

	1	2	3	4	5	
subitem not at all important	○	○	○	○	○	essential

Does your paper address subitem 21-ii?

Copy and paste relevant sections from the manuscript (include quotes in quotation marks "like this" to indicate direct quotes from your manuscript), or elaborate on this item by providing additional information not in the ms, or briefly explain why the item is not applicable/relevant for your study

Meine Antwort

OTHER INFORMATION

23) Registration number and name of trial registry

Ihre Antwort ist zu lang. Kürzen Sie einige Sätze in Ihrer Antwort.

Does your paper address CONSORT subitem 23? *

Copy and paste relevant sections from the manuscript (include quotes in quotation marks "like this" to indicate direct quotes from your manuscript), or elaborate on this item by providing additional information not in the ms, or briefly explain why the item is not applicable/relevant for your study

RCT #1: ClinicalTrials.gov NCT05858294, RCT #2: ClinicalTrials.gov NCT05987969

24) Where the full trial protocol can be accessed, if available

Does your paper address CONSORT subitem 24? *

Cite a Multimedia Appendix, other reference, or copy and paste relevant sections from the manuscript (include quotes in quotation marks "like this" to indicate direct quotes from your manuscript), or elaborate on this item by providing additional information not in the ms, or briefly explain why the item is not applicable/relevant for your study

Protocol see Supplementary Material.

25) Sources of funding and other support (such as supply of drugs), role of funders

Does your paper address CONSORT subitem 25? *

Copy and paste relevant sections from the manuscript (include quotes in quotation marks "like this" to indicate direct quotes from your manuscript), or elaborate on this item by providing additional information not in the ms, or briefly explain why the item is not applicable/relevant for your study

This study was sponsored and funded in full by Alena (Aya Technologies Ltd).

X27) Conflicts of Interest (not a CONSORT item)

Ihre Antwort ist zu lang. Kürzen Sie einige Sätze in Ihrer Antwort.

X27-i) State the relation of the study team towards the system being evaluated

In addition to the usual declaration of interests (financial or otherwise), also state the relation of the study team towards the system being evaluated, i.e., state if the authors/evaluators are distinct from or identical with the developers/sponsors of the intervention.

	1	2	3	4	5	
subitem not at all important	○	○	○	○	○	essential

Does your paper address subitem X27-i?

Copy and paste relevant sections from the manuscript (include quotes in quotation marks "like this" to indicate direct quotes from your manuscript), or elaborate on this item by providing additional information not in the ms, or briefly explain why the item is not applicable/relevant for your study

"Study authors MMG, JM, TM, SSM, SS, PS and AL were employed by Alena at the time of conducting the study and SL was paid on a consultancy basis. MMG, JM, SSM, SS, AL and MA own share options. QJMH is employed by University College London and acknowledges research grant funding from the Wellcome Trust, Carigest S.A. and Koa Health, and fees and share options for consultancies for Aya Technologies Ltd and Alto Neuroscience."

About the CONSORT EHEALTH checklist

As a result of using this checklist, did you make changes in your manuscript? *

- ○ yes, major changes
- ○ yes, minor changes
- ◉ no

Ihre Antwort ist zu lang. Kürzen Sie einige Sätze in Ihrer Antwort.

What were the most important changes you made as a result of using this checklist?

Meine Antwort

How much time did you spend on going through the checklist INCLUDING making changes in your manuscript *

I spent 3 hours doing this

As a result of using this checklist, do you think your manuscript has improved? *

- ○ yes
- ◉ no
- ○ Sonstiges:

Would you like to become involved in the CONSORT EHEALTH group?

This would involve for example becoming involved in participating in a workshop and writing an "Explanation and Elaboration" document

- ○ yes
- ◉ no
- ○ Sonstiges:

Auswahl löschen

Any other comments or questions on CONSORT EHEALTH

Please remove the "25 characters minimum" limit

Ihre Antwort ist zu lang. Kürzen Sie einige Sätze in Ihrer Antwort.

STOP - Save this form as PDF before you click submit

To generate a record that you filled in this form, we recommend to generate a PDF of this page (on a Mac, simply select "print" and then select "print as PDF") before you submit it.

When you submit your (revised) paper to JMIR, please upload the PDF as supplementary file.

Don't worry if some text in the textboxes is cut off, as we still have the complete information in our database. Thank you!

Final step: Click submit !

Click submit so we have your answers in our database!

Senden

Alle Eingaben löschen

Geben Sie niemals Passwörter über Google Formulare weiter.

Dieser Inhalt wurde nicht von Google erstellt und wird von Google auch nicht unterstützt. Missbrauch melden - Nutzungsbedingungen - Datenschutzerklärung

Google Formulare

Ihre Antwort ist zu lang. Kürzen Sie einige Sätze in Ihrer Antwort.

Ihre Antwort ist zu lang. Kürzen Sie einige Sätze in Ihrer Antwort.
